# Exploring the Role of the Rich Club in Network Control of Neurocognitive States

**DOI:** 10.1002/hbm.70485

**Published:** 2026-02-26

**Authors:** Alina N. Podschun, Richard F. Betzel, Urs Braun, Sebastian Markett

**Affiliations:** ^1^ Department of Psychology Humboldt‐Universität zu Berlin Berlin Germany; ^2^ International Psychoanalytic University Berlin Berlin Germany; ^3^ Department of Neuroscience University of Minnesota, Twin Cities Minneapolis Minnesota USA; ^4^ Masonic Institute for the Developing Brain University of Minnesota, Twin Cities Minneapolis Minnesota USA; ^5^ Department of Psychiatry and Psychotherapy Central Institute of Mental Health, Medical Faculty Mannheim, University of Heidelberg Mannheim Germany; ^6^ Hector Institute of Artificial Intelligence in Psychiatry Central Institute of Mental Health, Medical Faculty Mannheim, University of Heidelberg Mannheim Germany

## Abstract

The brain's rich club is a network of particularly densely interconnected regions, metabolically costly to maintain but central to the balance between functional segregation and integration. We assessed whether the rich club can accordingly be described as a control center of the brain, and present a systematic analysis of its involvement in maintenance of and traversal between various cognitively relevant functional states. Brain states were defined based on fMRI task‐evoked and resting‐state patterns of activity as provided by the Human Connectome Project (HCP). Using tools from network control theory (NCT), we computed the necessary effort needed for control of dynamics when the rich club, versus a size‐matched set of low‐degree peripheral regions, was prohibited from exerting control over dynamics. Control energy needed to traverse functional states was significantly higher, and stability of states significantly lower, when the set of peripheral regions was prohibited from control. Findings were stable across various rich‐club and null model definitions and across different parameter settings. A region's contribution to optimal control processes was instead associated with its affiliation with certain intrinsic connectivity networks and its position on the visual‐sensorimotor, but not sensory‐transmodal cortical gradient. We accordingly report that the rich club was systematically less involved in control of dynamics than the size‐matched set of peripheral regions. These results do not negate an integratory role of the rich club, but question its proposed role as a driver of control. Indeed, if it would inhabit such a role, we would have expected opposite results. Our findings fit with a position describing the rich club as a passive “data‐highway” which, by means of its high connectivity, can be easily controlled by peripheral regions and thus facilitate relevant communication channels between them. We call for methodological expansions of the control theoretical toolbox allowing for elaborations on the temporal dynamics of control processes.

## Graphical abstract

Using a network control theoretical framework, we found that the brain's rich club does not optimally control dynamics of the brain. Instead, size‐matched sets of random peripheral regions had a significantly higher impact on control metrics, questioning the rich club's proposed role as a control center of the human brain.

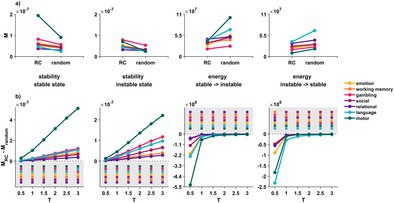

## 1 Introduction

The human brain possesses the remarkable ability to adapt to changing demands by transitioning between various functional states (Shine et al. 2016; Wu et al. 2021). These brain states are carefully stabilized to support the current task or goal. When circumstances shift, the brain adapts by transitioning into more appropriate activity patterns (Benisty et al. 2024; Braun et al. 2021; Gonzalez‐Castillo and Bandettini 2018). Brain states are characterized by recurring activity patterns distributed across the brain, arising from both physiological and cognitive processes (Greene et al. 2023). Importantly, these patterns are believed to have a direct influence on behavior (Benisty et al. 2024; Shine et al. 2016; Zink et al. 2021).

But where does this orchestration of neural activity originate? Emerging evidence suggests that the brain's underlying network structure constrains dynamics of brain states (Betzel et al. 2016; Cole et al. 2016; Honey et al. 2007; Sorrentino et al. 2021; Suárez et al. 2020). Various brain regions communicate through a vast network of nerve fibers, adhering to certain organizational principles (Hagmann et al. 2008; Sporns and Betzel 2016; Sporns and Zwi 2004; Stiso and Bassett 2018) that facilitate efficient, purposeful, and adaptive information processing (Rubinov 2016; Rubinov and Sporns 2010).

Furthermore, the brain's governance is a collaborative effort rather than a traditional centralized control center (Betzel et al. 2016; Kim et al. 2018; Zink et al. 2021). One essential feature contributing to network‐wide communication and integration is its rich club organization: This architectural feature involves well‐connected hub regions that preferentially link to each other, creating a densely interconnected subnetwork known as the “rich club” (van den Heuvel and Sporns 2011). Although maintaining the rich club is costly due to the need for long‐range and thick nerve fibers with high metabolic demand (Collin et al. 2014; van den Heuvel et al. 2012), it is most crucial for global integration and communication (de Reus et al. 2014; van den Heuvel et al. 2012).

Simulation studies have shown that such cortical hubs enable the brain to sustain a wide functional repertoire characterized by diverse dynamic configurations of peripheral regions (Bassett et al. 2013; Golos et al. 2015; Senden et al. 2014, 2017). These peripheral regions, with lower structural connectivity, interact with a stable high‐degree core facilitated by the rich club, supporting rapid information exchange among relevant peripheral regions in a task‐specific and goal‐directed manner while remaining attentive to the entire network (Senden et al. 2018).

Network control theory (NCT), initially developed in engineering, offers a mathematical framework to quantify the energy required to maintain brain states and control transitions between them (Gu et al. 2015; Karrer et al. 2020; Parkes et al. 2024). The energy required for optimal transitions is related to the cognitive demand of specific states (Cornblath et al. 2020), with more demanding target states, for example, states connected to a 2‐back working memory condition, being more energetically costly (Braun et al. 2021; Luppi et al. 2024). NCT may thus be a suitable tool to quantify cognitive effort and executive function, assess impairments due to illness or injury, and to determine possible treatment routes (Dimulescu et al. 2021; Hahn et al. 2023; Jeganathan et al. 2018; Medaglia 2019). Moreover, the application of NCT identifies specific brain regions likely to control relevant transitions, often implicating regions high in network communicability (Gu et al. 2017), and high‐degree regions within the rich club (Betzel et al. 2016; Braun et al. 2021). However, the specific role of the rich club in controlling transitions between behaviorally constrained brain states has yet to be explored.

In our current study, we leveraged NCT to investigate regional optimal control contributions to brain state transitions across seven different tasks. We hypothesized that the rich club plays a pivotal role in network control, exerting a more substantial influence on brain state stabilization and transitions than expected by chance.

## 2 Materials and Methods

### 2.1 Dataset

**FIGURE 1:**
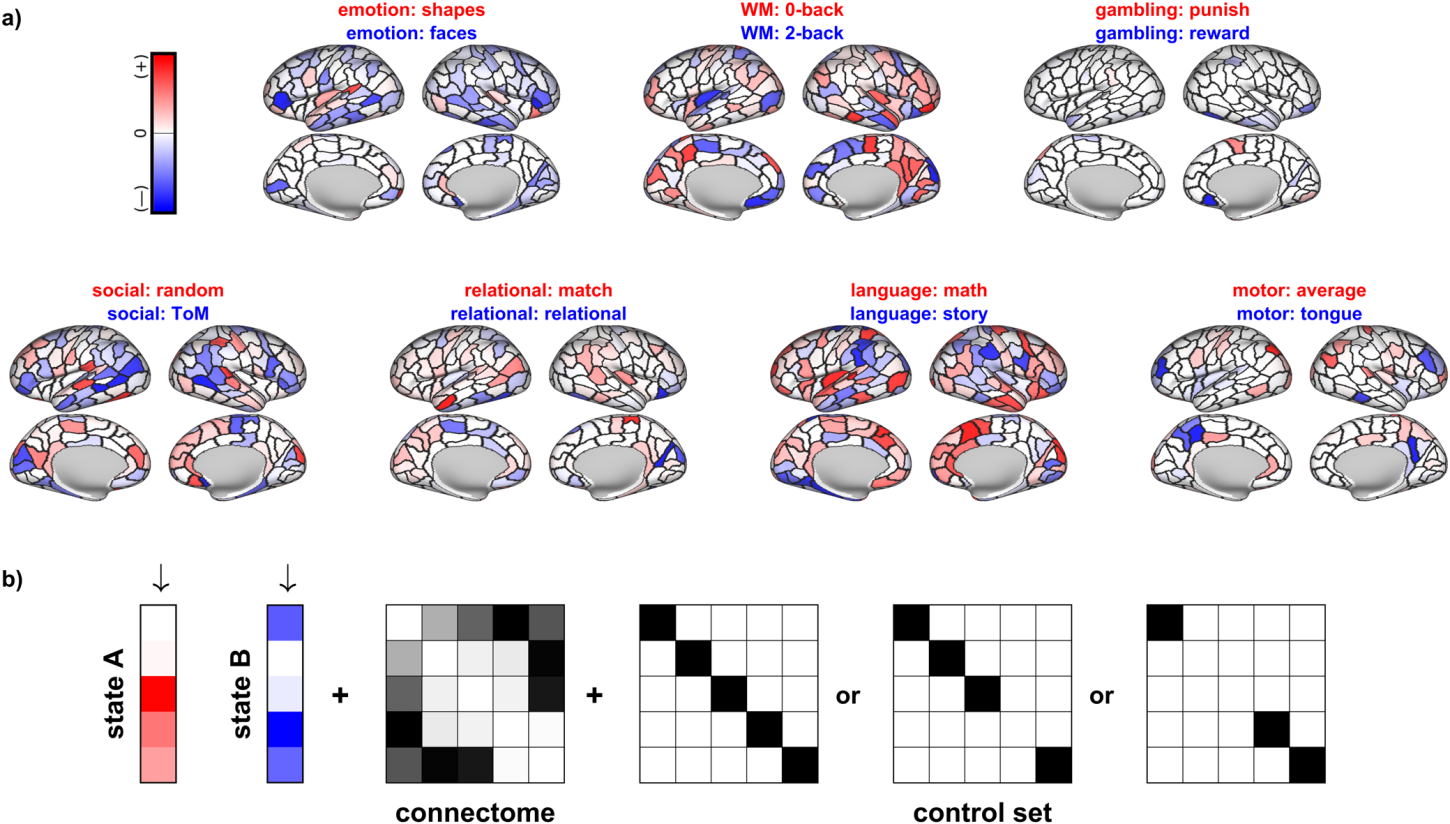
HCP brain states and overview of inputs into NCT analyses. We analyzed brain states constrained by the seven canonical HCP tasks. (a) Each behavioral task consisted of a more stable (significant activity in red, usually the control condition) and a less stable brain state (significant activity in blue, usually the experimental condition). The more saturated the color, the higher the difference in activation between the two states. Figure 3 shows results from corresponding NCT analyses. (b) Optimal control analyses require various inputs, here shown in a toy network consisting of five nodes. For each participant, trajectories between the two behaviorally constricted brain states were calculated constrained by a participant‐specific, FA‐weighted and undirected connectome and given a specific control matrix that specifies to which regions control is restricted. We investigated global control (all regions are allowed to exhibit control), regional control (all but one region is allowed to exhibit control) and also investigated the role of a subset of brain regions, excluding the rich club or a size‐matched reference set from control. Given these components and penalizing as well as time parameters, control theoretical formulae can be solved for an optimized amount of control energy needed to traverse from the initial to the target state (see Section 2).

Data were drawn from the Human Connectome Project (HCP), specifically from the subset of 100 genetically unrelated participants. The HCP provides data from seven tasks: an emotion matching task, a version of an *n*‐back working memory task, a gambling task, a language task, a motor task eliciting movement, a relational task requiring participants to match visual properties of objects, and a social task assessing whether participants think of objects as having a random or social interaction. We used preprocessed (Glasser et al. 2013; Van Essen et al. 2013) first‐level contrasts from all seven tasks (see Figure 1). See Barch et al. (2013) for an overview of the HCP's choice of task paradigms.

Task data was combined with preprocessed diffusion‐weighted MRI data as provided by the HCP (see Van Essen et al. 2013 regarding acquisition pipeline) to reconstruct participant‐specific connectomes. The Supporting Information contains more detailed descriptions of the MRI acquisition pipeline, (pre‐)processing steps, and the task paradigms.

### 2.2 Connectome Reconstruction

We processed the structural data using the connectivity reconstruction toolbox CATO (de Lange et al. 2023), including application of CATO's minimal preprocessing pipeline to create cortical parcellations from the Freesurfer output. Cortical parcellations were created based on the Lausanne subparcellation of the Desikan–Killiany atlas (Cammoun et al. 2012), resulting in 219 cortical regions. White matter fiber organization was estimated using a combined approach of diffusion tensor imaging (DTI) and generalized *Q*‐sampling imaging (GQI), leveraging the strengths of both methods. DTI modeling was here performed in voxels with one peak, GQI in those with multiple peaks (de Lange et al. 2023). Fiber tracking in CATO is based on a version of the deterministic “Fiber Assignment by Continuous Tracking” (FACT) algorithm originally described by Mori et al. (1999); see de Lange et al. (2023) for details regarding the procedure. For any two regions in the parcellation, we determined the shortest fiber segment (if any) and calculated the mean fractional anisotropy (FA) of all voxels in each fiber segment, weighted by the length of the traversed path through each voxel. CATO allows for filtering of fibers based on a maximal angle allowed for turns (here: 45°), minimal FA value (here: FA ≥ 0.1) or maximal fiber radius (here: 500 mm). Fibers violating any of these criteria were discarded. The resulting information was then used to reconstruct weighted structural networks for each participant. While FA‐weighted connectomes were used throughout the main analysis, we additionally derived unweighted connectomes (with any connection identified as present set to 1, and all others set to 0) and connectomes weighted by the number of streamlines (NOS) for parameter exploration. Of the initial *N* = 100, *N* = 98 participants (*n* = 45 male, *n* = 53 female; mode of age = 33) were included in our investigation: Following the procedure described in Van Den Heuvel et al. (2019), specifically the subsection “Outliers” (p. 5) we tested for outliers based on a group average prevalence matrix characterizing the average number of times a connection between two nodes was observed across participants. We excluded data of two participants whose connectome maps significantly deviated from the group average (i.e., scored 2× the interquartile range [IQR] above or below the first or third quartile of total connection prevalence in the group). All analyses used the full set of *N* = 98 participants, except for the exploratory analyses with NOS‐weighted connectomes, where one additional participant with outlier values was excluded (see Figure S4), resulting in a set of *N* = 97 for these analyses.

Main results were also replicated on a commonly used and biologically plausible (Van Essen et al. 2013; Yan et al. 2023) parcellation, namely the multimodal parcellation provided by the HCP. Details on connectome reconstruction for these replicatory analyses are provided with the Supporting Information.

### 2.3 Brain State Definition

We based brain states on the two main experimental contrasts for all tasks; regarding the motor task, we opted for those contrasts that contained data from both hemispheres. Individual cortical parcellations were brought into CIFTI format using Freesurfer and connectome workbench (Marcus et al. 2011), and then used to parcellate functional data, allowing us to respect individual differences in regional boundaries. We defined regional activity values by calculating mean activation over time and per region, resulting in two Lausanne‐parcellated brain states per task and participant (see Figure 1).

Our main analyses focus on controlling transitions between pairs of brain states that belong to the same task: Behavioral tasks are designed to elicit similar brain states that differ primarily in a critical variable, such as working memory load or emotional valence. This design choice guarantees a high level of experimental control but may limit the generalizability and ecological validity of our findings, as the brain often transitions between more diverse activity patterns in real‐life scenarios. To approximate more diverse transitions, we additionally analyzed the most representative state of each task (e.g., high working memory load in the working memory task, or emotional valence in the emotion task) and investigated transitions *across* tasks.

To further increase the ecological validity of our results, the Supporting Information also contains replication of our main analyses based on brain states extracted from resting‐state instead of task‐based activity data (see also Figure S6).

Visualizations using brain projections in this study are always created using the connectome workbench.

### 2.4 Definition of the Rich Club

**FIGURE 2:**
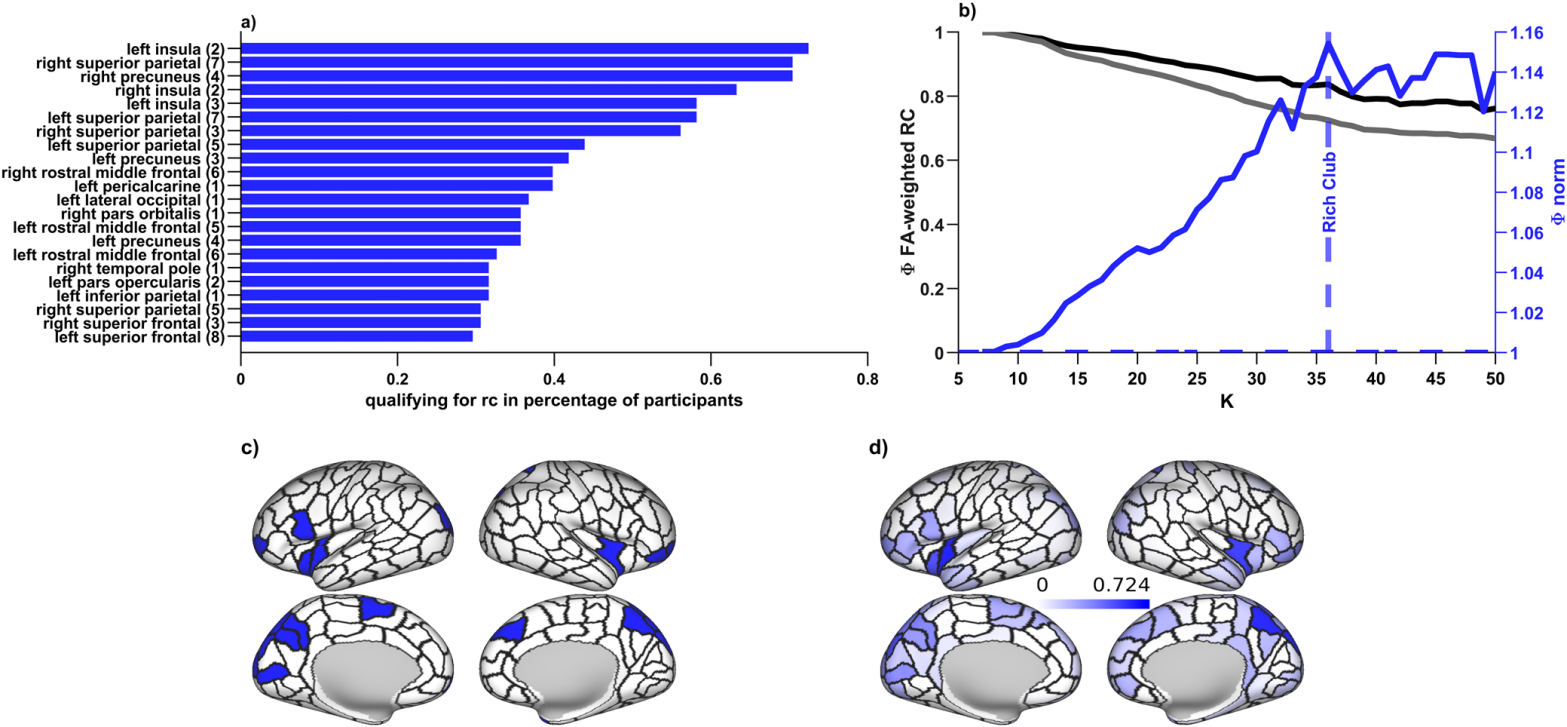
Rich‐clubness and the group‐level rich club. (a) 22 regions were qualifying for the group‐level rich club and are shown here; the corresponding relative frequency with which a given region was assigned rich club membership on a participant‐specific level is indicated in the bars and varied greatly. (b) The rich club was defined by comparing the structural connectivity of the empirical network (black) in relation to a randomized network (gray) for every nodal degree level k. Comparison of the two leads to a normalized (blue) rich club curve, which peaks at $k_{\max\left(\Phi \right)}$. This peak characterizes the degree level k at which connectivity in the empirical network is maximally stronger than expected by chance, and the peak within the significant rich club regime was set as the starting point of the rich club, with all regions with nodal degree greater than $k_{\max\left(\Phi \right)}$ being considered rich club members. Curves are based on a group‐level connectome and included merely for visualization. (c) This procedure led to 22 group‐level rich club members (see also a), which are here projected onto an inflated map of the cortical surface. (d) There was considerable interindividual variation with which a region would be assigned rich club status on a participant‐specific level. Saturation of the color indicates the relative frequency with which a region would be assigned rich club membership on an individual level; the 22 regions for which this was most frequently the case constitute the group level rich club shown in (a) and (c).

We established a rich club regime following the procedure detailed in Riedel et al. (2022). Using the Brain Connectivity Toolbox (BCT; Rubinov and Sporns 2010; version 2019_03_03), we first computed weighted, i.e., nodal strength‐based rich club coefficients for our empirical connectomes (rich_club_wu.m), and then for 10,000 random brain networks, each connection randomly rewired 10 times while preserving degree distribution (randmio_und.m). The normalized rich club coefficient Φ for each level *k* of the degree distribution is the ratio of the empirical coefficient over the random coefficient, averaged across all 10,000 iterations. Setting a cut‐off at $k_{\max\left(\Phi \right)}$ within a participant's significant rich club regime (see Figure 2b), on average 10.10% of an individual's nodes (around 22 nodes) were considered rich club members. Group‐level rich club definition was based on the 10.10% top ranking nodes across participants, that is, those 10.10% of nodes that qualified most consistently as rich club members across participants, resulting in 22 regions constituting the group‐level rich club (see Figure 2a,c,d). Individually specific rich clubs were subsequently based on the 22 top degree nodes of an individual, ensuring that while the exact composition of the rich club was participant‐specific, the number of rich club members remained matched across individuals. We repeated main group‐level analyses on an individual‐specific level. Additionally, we include group‐level replications of our main analyses on a different widely used parcellation scheme in the Supporting Information (see also Figure S5).

For inferential evaluations, we compared results from excluding the rich club regions from the set of control nodes to a null model in which we excluded a size‐matched set of semirandomly selected regions instead. Our null models and accordingly the semirandom selection of regions are based on a spin model paradigm (Alexander‐Bloch et al. 2018) more closely detailed in the subsection on statistics.

### 2.5 Control Energy and Stability

The control energy framework employed here is a linear approximation of brain dynamics based on (a) a stabilized structural connectivity matrix S of a participant, (b) two vectors $x\left(t\right)$ describing brain states at a given point in time, (c) a control matrix C delineating which regions are allowed to control brain dynamics, and (d) a vector of control energy $u\left(t\right)$ representing the control input into the control nodes at a given point in time (Braun et al. 2021; see also Betzel et al. 2016; Karrer et al. 2020; and see Figure 1b):

(1) $$y\left(t+1\right)=\mathrm{Sx}\left(t\right)+\mathrm{Cu}\left(t\right)$$

This formula can be solved for an optimized value of parameter u minimizing both required control energy as well as length of trajectory between the initial and target state through state space (Braun et al. 2021; Karrer et al. 2020). We define optimal control energy for the transition between two distinct brain states as squared integral of a region's energy contribution over time, averaged across all 219 brain regions. We define stability of a state accordingly, as the optimal control effort needed for maintenance of a state, i.e., as the inverse of the squared integral of a region's energy contribution over time, averaged across all 219 brain regions, when defining the same state as initial and target state (Braun et al. 2021). Importantly, since initial and target state are identical for analyses of brain state stability, but different for analyses of the control energy needed for transitions, both metrics reflect unique and distinct control processes. All NCT analyses were implemented in MATLAB R2021b, based on NCT‐functions publicly provided by Braun et al. (2021), especially optim_fun.m, available under https://github.com/ursbraun/network_control_and_dopamine. Within our main analyses, we set the parameters for time horizon T=1 and penalization parameter ρ=1; however, we also systematically investigate the impact of changing the values for time horizon T. We transformed raw NCT output by dividing by the number of timesteps given by our time horizon T, allowing for comparisons across different settings of the time parameter.

The control energy framework was applied with three distinct manipulations of the control matrix C (see Figure 1b). In order to probe the general optimal control energy and stability, we included all 219 regions into the control set, enabling all regions to contribute to state transitions and maintenance.

To probe the role of the rich club, we utilized a reduced set of control nodes. We either excluded rich club regions from the set of control nodes by setting their respective entries in the control matrix to zero, or excluded a size‐matched set of random regions from the control set. Please note that removing nodes from the control set does not remove them from the connectome; they can still be influenced and controlled by other nodes, but cannot exert control themselves (Betzel et al. 2016).

In order to probe individual contributions of brain regions, we removed single brain regions from the control set and evaluated the change in control energy and stability against the full control set. Regions were then rank‐ordered according to their impact on stability and control energy metrics, and each region's mean rank across tasks and across stability and energy measures was later correlated with a regional property of interest. We used product–moment correlation coefficients to quantify the relationship between regional control contribution and regional specifics.

All network control theoretical analyses in this study are conducted within an optimal control framework. Importantly, optimal control analyses are concerned with control processes between two *specific* pre‐defined brain states. They accordingly do not investigate a more *general ability* of the brain for control, and are thus contrasting with network control analyses that are concerned with the brain's average or modal controllability. Analyses of (optimal) control energy are particularly suited toward investigations of specific cognitive functions or for control of cognitively relevant states (Parkes et al. 2024), and are thus employed here. We refer the interested reader to work by Karrer et al. (2020) or Parkes et al. (2024) for an in‐depth discussion of the differences between controllability and control energy. Code to conduct NCT analyses as in this study is accessible at: https://github.com/markett‐lab/NetworkControlRichClub.

### 2.6 Regional Characteristics

When investigating the relationship between individual regions' control roles and other regional characteristics, we associated a region's mean rank in control contribution with various other regionally specific properties (specifically, the mean of that variable per node and across participants). We here focused on possible associations with a region's participation coefficient, regional communicability, a region's membership in specific resting state networks, a region's position on various cortical gradients and lastly, the extent to which structure and function were coupled in a region.

Previous work has highlighted the importance of peripheral over core regions for information integration and fast processing (Betzel et al. 2016; Gollo et al. 2015), for instance by proposing the concept of a “diverse club” of regions with the most diverse connections across functional modules in the connectome (Bertolero et al. 2017; Lohia et al. 2024). Using the canonical 17‐network division of the cerebral cortex (Yeo et al. 2011), and the BCT function participation_coef.m, we calculated each region's mean participation coefficient across participants and investigated its correlation with the regional rank measure. Importantly, this is an extension of the approach in Bertolero et al. (2017), where functional modules and edges were derived from participants' functional connectivity matrices. Instead, we methodologically extend their investigations by continuing to use our fiber‐tracking derived structural edges and combining them with modules based on well‐established resting state networks (which are themselves underpinned by structural connectivity; Hermundstad et al. 2013; Honey et al. 2009).

While the structural network itself encodes direct connections between nodes, brain regions can also communicate via indirect paths. We thus follow Betzel et al. (2016) in calculating communicability as the weighted sum of paths of all lengths between two brain regions, such that high communicability of a region reflects relative ease to communicate with other regions either via direct or indirect connections. Possible associations between nodal communicability and nodal control contribution were then also assessed to reflect the chance that nodes might be top controllers not merely based on direct but also indirect connections.

We also investigated whether a region's membership in a specific resting state network could be connected to its control role, and again used the 17‐network division of the cerebral cortex by Yeo et al. (2011) to this end. We assigned every brain region to one of the 17 functional networks based on spatial overlap at the vertex level, and then computed the mean regional rank per network, afterwards assessing whether the mean control contribution of specific networks was significantly higher than that of others.

Additionally, we explored two other potential relationships: On the one hand, possible associations between a region's control contribution and its position on intrinsic cortical gradients extractable from resting state functional connectivity patterns (Margulies et al. 2016). On the other hand, possible associations of regional control with the extent to which structure and function are coupled in a region (Valk et al. 2022). Regarding cortical gradients, we correlated a region's position on well‐established cortical axes that (a) separate more sensory regions from more transmodal regions and (b) more visual from more sensorimotor regions (Margulies et al. 2016). Regarding possible structure–function coupling, we followed the approach in Valk et al. (2022) and relied on data provided by the BrainSpace toolbox (available under https://github.com/MICA‐MNI/BrainSpace) to calculate region‐specific metrics reflecting how closely a region's functional connectivity profile was coupled to its microstructural profile. We then, again, assessed correlations of this coupling metric with a region's control contribution.

### 2.7 Null Models and Statistics

Inferential analyses are based on null models created via a spin‐test paradigm (Alexander‐Bloch et al. 2018; Váša et al. 2018; code available under https://github.com/frantisekvasa/rotate_parcellation) which randomly rotates spherical surface projections of the atlas while preserving hemispheric symmetry and contiguity. For NCT analyses of the rich club as a network, this procedure was repeated 50 times, resulting in 50 rotated brain maps. We used various null model strategies to corroborate results: In the minimal configuration, we simply size‐matched the set of null‐model regions to the rich club, and excluded 22 regions across the 50 null model maps from exerting control. We then statistically compared results from NCT analyses to the results from instead excluding the rich club from the matrix of control nodes. However, rich club regions are inherently more interconnected than peripheral regions (van den Heuvel and Sporns 2011) and their connectivity profiles are more similar to each other than those of a size‐matched set of random regions. To ensure that our results were not spuriously inflated by the null model simply removing regions with more mutual connections or more diverse connectivity profiles from the control set, we repeated analyses at the participant‐level: This time, however, we constrained the random selection of reference regions to match the rich club most closely regarding the similarity of their connectivity profile or the sum of their mutual connections. In this way, we ensure that any significance of our results is not merely attributable to possible differences in interconnectedness or connectivity profiles between the rich club and our null model regions.

All statistical analyses of differences between rich club and null model metrics were conducted via repeated measures analysis of variance (rANOVA), and separately for all NCT metrics and pairs of brain states. NCT results when excluding the rich club versus mean NCT results across the 50 null models were considered as repeated measurements. In line with previous research (Braun et al. 2021) we additionally controlled for mean nodal activity difference between the initial and target brain state, to ensure that possible differences in control metrics were not merely reflecting differences in brain activation. Mean activation per ROI for each participant and brain state was extracted, and the difference in mean regional activation between the initial and target state was included in the repeated measures model as a covariate. The statistical model can be described by the following formula:

(2) NCT∼RC/random+CV

where NCT refers to the stability or control energy metric, RC/random captures whether the contribution is from the rich club or reference set, and CV refers to the mean regional activation difference as a covariate. Post hoc one‐tailed paired sample *t*‐tests were conducted to confirm the direction of significant main effects.

Additionally to using the spin test model when considering control contributions of networks of regions, we applied the same approach to evaluate correlations of various region‐specific properties (nodal degree, participation coefficient, communicability, membership in functional networks, as well as the position on cortical gradients) and regional control energy. Regional specifics of interest were here permuted 10,000 times via the spin‐test approach to construct null distributions against which the empirical values were compared. *p*‐Values were assigned by counting how often the empirical value was exceeded by permuted values and dividing the resulting number by 10,000.

## 3 Results

We studied the stability of brain states during seven canonical behavioral tasks from the HCP (Van Essen et al. 2013; see Figure 1), and the control energy required to transition between these states. Using FA‐weighted, undirected structural brain networks, we employed NCT to approximate linear brain state dynamics and the optimal control framework to determine the required control energy to maintain and switch between these task‐constrained brain states.

### 3.1 Global and Regional Control Energy and Stability

**FIGURE 3:**
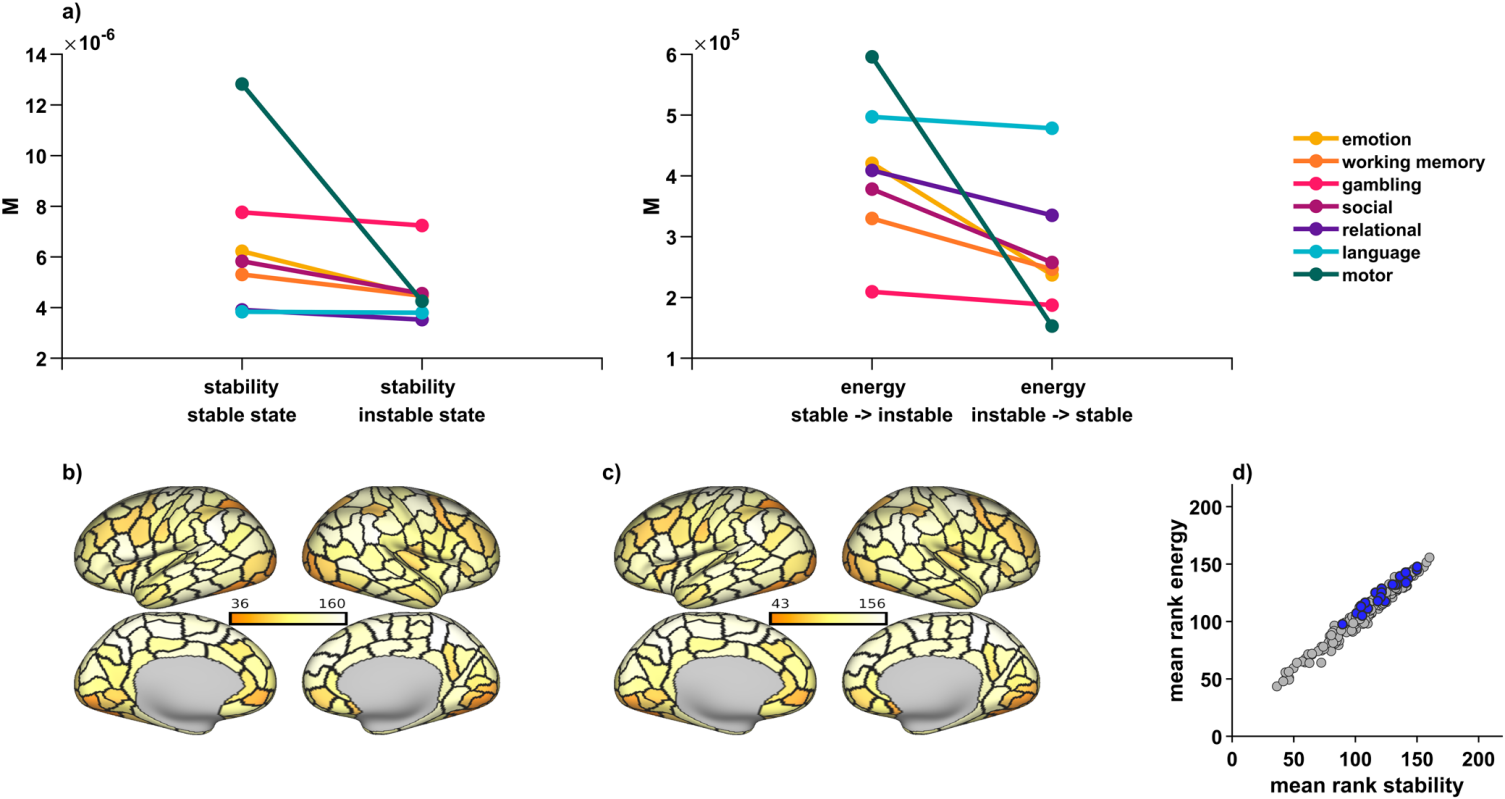
Results from global and regional control analyses—a subset of regions is task‐generally implicated in control. We investigated global control of brain state trajectories, as well as possible task‐general control roles of specific regions. (a) For every task except for the language task, there was one state that was significantly more stable than the other state (left panel). Transitions from the stable state into the instable state were associated with significantly higher control energy needs than the reverse, again except for the language task (right panel). Displayed are mean global NCT values across individuals and for each measure. (b and c) A subset of regions qualified as top controllers across tasks and was implicated both in the maintenance of brain states (dark colors in b), as well as in the control of brain state transitions (dark colors in c). Another subset of nodes task‐overarchingly did not contribute to regional control of brain states (light colors in b and c). Plotted is a region's mean rank in regional control contribution across participants and tasks, with dark colors indicating low ranks and thus high control contribution, light colors indicating high ranks and thus low control contribution. Black borders indicate different regions as defined in the Lausanne atlas. The Supporting Information contains corresponding task‐ and measure‐specific plots (Figure S1). (d) Shows the strong relationship between a region's contribution to stability and energy measures. Rich club regions are indicated in blue; they overlapped with a set of comparatively high‐ranking, i.e., not impactful, regions.

Each behavioral task is designed to elicit a minimum of two distinct brain states (see Figure 1). We first examined the global stability of all 14 brain states and the control energy needed to transition between the two states within each task, considering all brain regions as possible controllers (see Figure 1b, leftmost control set). Except for the language task, the two brain states within each task differed significantly in their stability (all p<0.01, Figure 3a). Transitions from the more stable to the less stable state required significantly more control energy than the reverse, again with the exception of the language task (all p<0.05). For the language task, the math condition was on average more stable than the language condition, and is thus reflected as the more stable contrast in figures. Importantly, this is for visualization purposes only, as for this specific task, the difference in average stability between conditions did not reach significance. Full rANOVA results can be found in the Supporting Information (Tables S1 and S2).

In a second step, we quantified each individual brain region's contribution to maintaining and transitioning between brain states (see Figure 1b, middle control set). Brain regions contributing to task‐general brain state maintenance also facilitated task‐general brain state transitions (Figure 3b–d). For stability measures, we observed mean ranks between ${\mathrm{rank}}_{\min}=36$ and ${\mathrm{rank}}_{\max}=160$ across tasks. For transition measures, we observed mean ranks between ${\mathrm{rank}}_{\min}=43$ and ${\mathrm{rank}}_{\max}=156$ across tasks. With lower ranks indicating higher control impact, minima values show that while some regions had more task‐general contributions than others, there was still considerable variation across tasks and measures in top control nodes. Maxima values, however, point to a subset of nodes that did consistently not contribute to the control of brain states across tasks (see also Table S23).

### 3.2 Regional Control Energy and Stability of Rich Club Regions

We identified 22 group‐level rich club regions, which were most consistently assigned to the rich club based on the maximal normalized rich club coefficient at the participant‐level (Riedel et al. 2022). These regions included the superior parietal cortex, insula, precuneus, rostral middle frontal cortex, superior frontal cortex bilaterally as well as *left* pericalcarine cortex, lateral occipital cortex, pars opercularis, inferior parietal cortex, and *right* pars orbitalis and temporal pole (Figure 2a,c). Considering the control contribution of individual regions, rich club regions spatially overlapped with middle to high rank on the task‐general control metric (Figure 3d, compare also Figure 2c to Figure 3b,c), and had significantly higher ranks for both stability ($M_{\mathrm{rank}}=134;p=0.0275$) and energy ($M_{\mathrm{rank}}=147;p=0.0021$) than expected by chance (10,000 permutations), indicating that rich club regions indeed contributed less to stability and control energy than the periphery.

In order to ensure that this observation was not confounded by the strict binary division of brain regions into a rich cub and a periphery (Alstott et al. 2014; Griffa and van den Heuvel 2018), we additionally correlated regional nodal degree with the regional ranks, again comparing the resulting correlation to a null distribution of 10,000 permutations using a spin test. Nodal degree correlated positively with a region's rank for both stability (r=0.35;p<0.001) and control energy (r=0.42;p<0.001) measures, confirming high degree regions contributed less to both state maintenance as well as transitions between states.

While our group‐level rich club included those 22 regions most frequently considered rich club members in participant‐level analyses, there was still considerable variation in the relative frequency by which a region would be assigned to the rich club (Figure 2d). Importantly, replication on individual‐level rich club definitions confirmed all group‐level results: None of the participants exhibited a significant involvement of rich club regions in stabilizing or transitioning between brain states. In the majority of participants, rich club regions contributed significantly less to both brain state stability (90.82%) and transitions (72.45%) than expected by chance. For a subset of participants with comparatively low‐ranking (i.e., comparatively impactful) rich clubs, findings were nonsignificant; for this subset of participants, rich club nodes and peripheral nodes did not differ in their control contribution (see Figure S2).

### 3.3 Control Energy and Stability of the Rich Club as a Network

**FIGURE 4:**
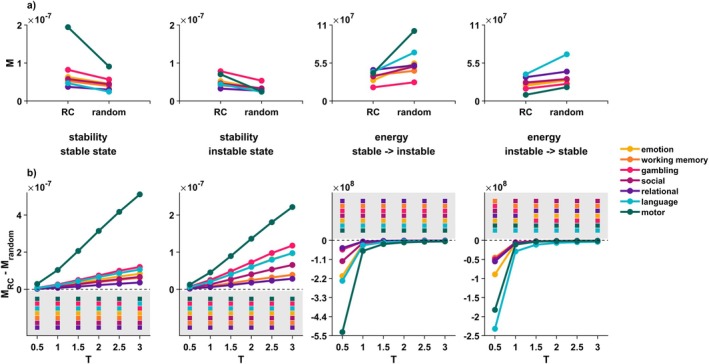
The rich club is significantly not implicated in brain state control. The rich club always contributed less to maintenance of brain states and transitions between them than a size‐matched reference set of randomly chosen regions. Displayed are mean NCT values across individuals, and for each measure and task. (a) A brain state was always less stable when excluding random regions from the set of control nodes than when excluding the rich club. Additionally, when excluding random regions, more energy was needed to traverse between brain states than when excluding the rich club. Repeated measures rANOVA found all differences to be significant for all tasks except for one state transition in the relational task, with all other p<0.01. (b) The direction of this effect remained the same when varying the length of the time horizon *T* over which trajectories were allowed to evolve; for stability, the difference between excluding the rich club vs. random regions became even more pronounced for higher *T*. For energy, the difference became less pronounced; importantly, the direction of the effect remained the same and the difference remained significant except for most state transitions within the relational task. *M* = mean value across participants, *T* = parameter choice for time horizon *T*.

We further corroborated our finding through optimal control analyses in which we excluded all rich club regions from the matrix of possible control regions, in effect considering a possible control role of the rich club as a network (see Figure 1b, rightmost control set). This exclusion resulted in lower control energy requirements for stabilizing (all p<0.05, Figure 4) and transitioning between brain states (p<0.05 for all but one state transition for the relational task, Figure 4a), compared to excluding a random group of 22 regions (spin test with 50 permutations). While the difference between the control role of the rich club and reference set was not significant for one state transition in the relational task, the direction of effect still remained the same: The rich club did not contribute more to control of this state transition than the reference set, only it also didn't contribute significantly less. These findings again confirm that—compared to any other group of brain regions—the rich club contributed less to the stabilization of brain states and less to the controlled transitions between states (see Tables S3 and S4 for detailed statistics). We were additionally able to replicate these results on a different cortical parcellation and using a different connectome reconstruction toolbox, which we detail in Figure S5.

### 3.4 Replication on Additional Connectome Weighting Schemes and Parameter Settings

The optimal control framework within NCT evaluates brain state transitions and maintenance over time, specified by the parameter T (time horizon). To ensure the robustness of our findings regardless of the chosen time horizon, we systematically varied the parameter and re‐conducted our analysis. Our main finding remained consistent across all time horizons except for 7 out of 12 state transitions within the relational task (all other p<0.001, Tables S5–S8). Again, while not reaching significance in these state transitions, the direction of effect nonetheless remained the same: the rich club never contributed more than the reference set. Notably, control energy estimates were less sensitive to the removal of rich club regions compared to random regions for longer time horizons, while stability estimates were more sensitive to rich club removal (see Figure 4b).

Furthermore, we explored the impact of different connectome weighting approaches by comparing our original results based on FA‐weighted connectomes with results obtained with connection weights according to the number of streamlines (NOS) as well as with a binary, unweighted network. The results remained significant for the NOS‐based connectome (all p<0.05, Tables S9 and S10). When employing a binary matrix, however, results became less consistent: We observed significant main effects for all language and motor task measures (all p<0.001), but in the other five tasks, most energy (save for one state transition in the emotion and relational task) and most stability measures (save for the instable states of the emotion and gambling task) failed to reach significance (refer to Tables S13 and S14 and Figure S3).

### 3.5 Replication on Participant‐Level Rich Club and Reference Set Definitions

**FIGURE 5:**
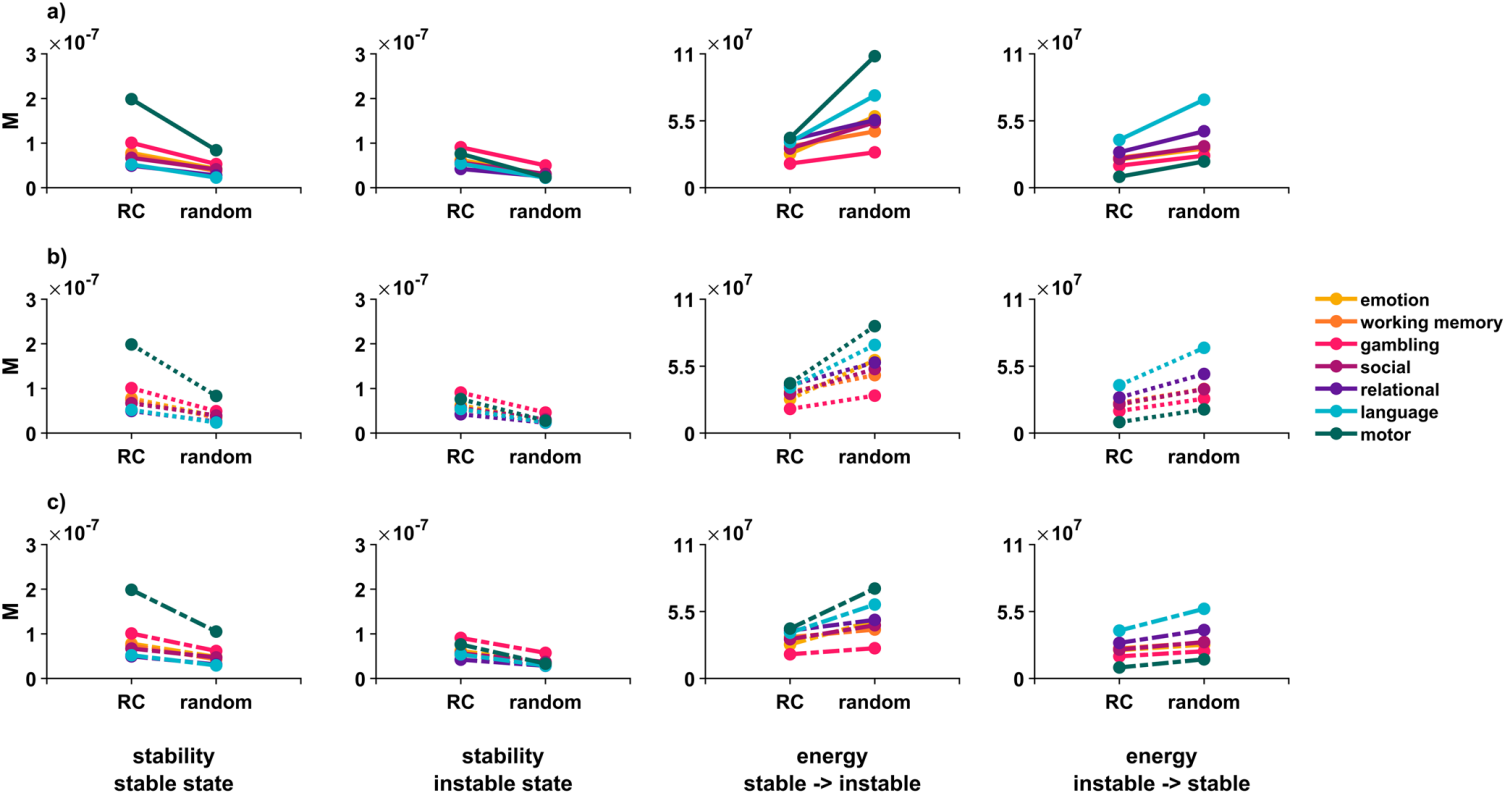
The rich‐club does not control state transitions when individual variation in membership and different definitions of the reference set are taken into account. We have reported considerable individual variation in rich club members when the rich club was defined on a participant‐specific level (see Figure 2). We thus repeated analyses, selecting the 22 rich club regions individually for each participant and re‐calculating the impact of prohibiting those regions from exerting control versus prohibiting a size‐matched reference set. Displayed are mean NCT values across individuals, for each measure, and for various sets of random regions. (a) Solid line plots show the results of individual level analyses when the set of random regions was selected in the same manner as in the group level analyses, that is, fully based on the spin‐test procedure described in the main text and methods section. We were able to fully replicate the effect found for a group‐level rich club definition. (b) Dotted plots show results of individual level analyses when the set of random regions was selected in a way in which the similarity of its members' connectivity profiles most closely resembled that of the individual's rich club. Again, we were able to fully replicate the group‐level effect. (c) Dash‐dotted plots show results of individual level analyses when the set of random regions was selected in a way in which the sum of its connections most closely resembled that of the individual's rich club. We were able to replicate the group‐level effect for all stability measures and all but three state transitions; for these three transitions, differences between exclusion of the rich club and the reference set were nonsignificant. *M* = mean value across participants, RC = rich club.

To account for the considerable interindividual variability with which regions were assigned rich club status on an individual level, we also repeated the control set exclusions of the network of rich club regions using participant‐specific rich club definitions. Despite the considerable interindividual variability in rich club definitions, all differences relative to a random reference set remained significant (see Figure 5a and Tables S15 and S16).

We also used alternative additional constraints to our null models: Differences in stability and control energy remained significant in all cases where we compared the rich club to a connectivity‐profile matched reference set (Figure 5b). When comparing the rich club to a connection‐sum‐matched reference set (Figure 5c), all differences remained significant for all stability measures and all but three state transitions, all of which concerned transitions from the instable to stable state (see Tables S17–S20).

### 3.6 Replication on Between‐Task Transitions

**FIGURE 6:**
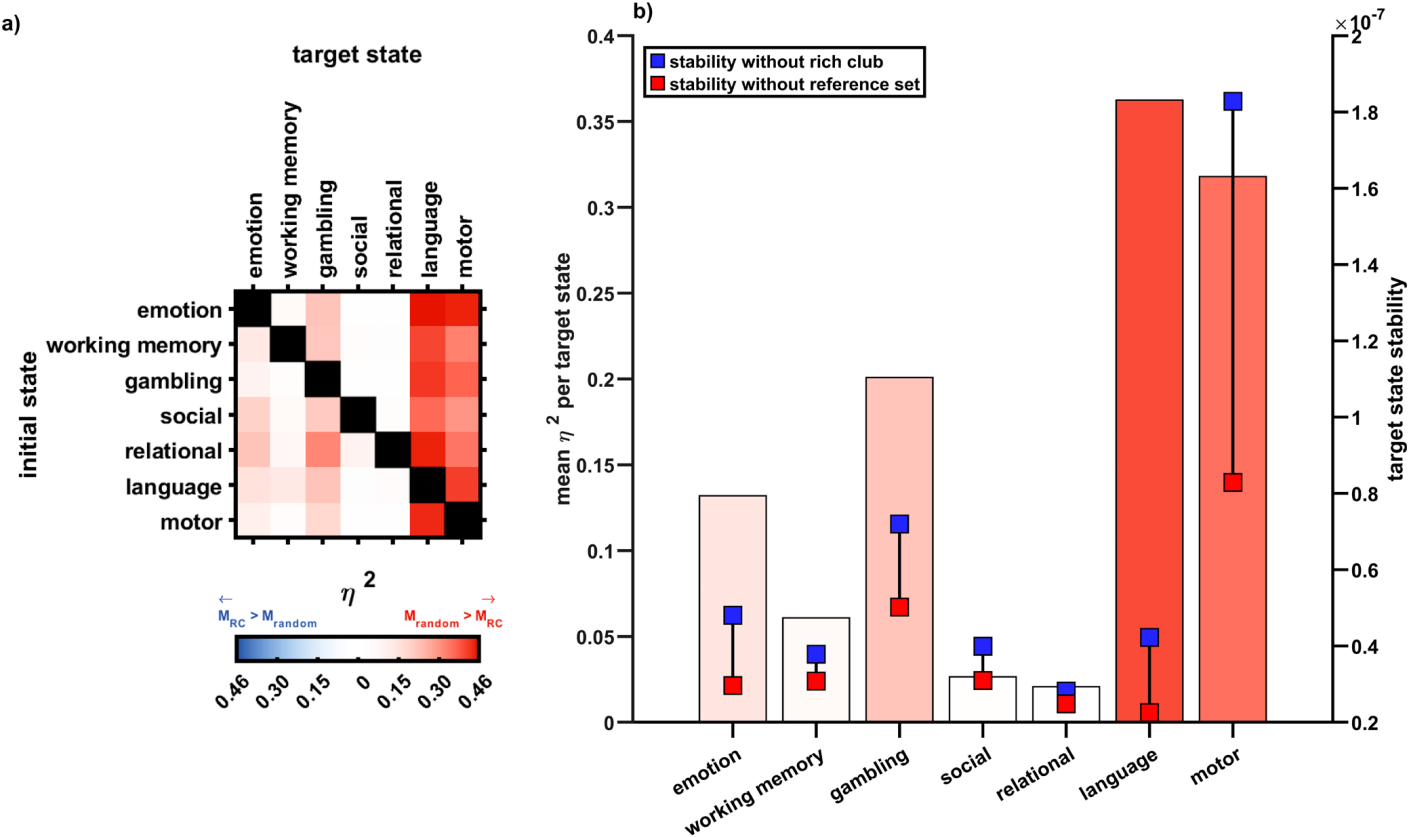
The rich club does not control brain state transitions across tasks; effect sizes depend on the target state. In reality, the brain often traverses between states that are very diverse; arguably more diverse than two states designed for one and the same experimental paradigm. We approximated this diversity by repeating our analyses for transitions across tasks, that is, from the typical brain state associated with one paradigm to that associated with a different paradigm altogether. Shown are effect sizes for the difference in control metrics when excluding the rich club vs. when excluding random regions from control. The more saturated the color, the higher the effect size. (a) Repeating our findings from within‐task transitions, the periphery instead of the rich club dominated transitions across tasks: Red colors indicate a more drastic impact when excluding random regions; blue colors indicate a more drastic impact when excluding the rich club. This effect was particularly stark when the target state to be transitioned into was associated with the language or motor task. (b) The role of the periphery in controlling state transitions was particularly stark for cases in which it was, compared to the rich club, increasingly involved in stabilizing the target state: Bars show mean effect sizes of between‐task transitions per target state (left *y*‐axis). Blue and red data points show stability of the respective state when excluding rich club regions (blue) vs. a random reference set (red) from control (right *y*‐axis).

So far, we have focused on *within*‐task transitions, examining the impact of exclusions on state stabilization and state transitions within a given behavioral task. For *between*‐task transitions, we again found that removing the rich club from the control set had a significantly reduced impact on brain state stability and brain state transitions compared to removing a size‐matched reference set of random regions (see Figure 6). Effect sizes and significance of this finding varied by target state. Peripheral regions played a more critical role in control of transitions when the target state was the representative language or motor state (Figure 6); but were not differentially relevant to the rich club for some transitions toward the representative social, relational or working memory state (Figure 6 and Table S21). The mean effect size of transitions toward a target state correlated positively with the difference in that state's stability when excluding the random reference set versus the rich club from the set of control nodes, but this effect failed to reach significance (r=0.64; p=0.0659, see also Figure 6b). Importantly, we also didn't find significant correlations between effect size and the stability measure when prohibiting the rich club (r=0.57, p=0.0911) or when prohibiting the reference set (r=0.50, p=0.1541) from control.

We also conducted analyses on brain states extracted from resting state data, offering another approach aimed at increased ecological validity of our findings. Detailed results on this replication can be found in Supporting Information and in Figure S6, where we show that results are overwhelmingly replicable on brain states defined in this alternative manner. In summary, the removal of the rich club from the control set had less of an impact on brain state control than the removal of a similar group of random regions. This observation was consistent across behavioral tasks, across transitions, across rich‐club definitions, across NCT parameter settings, and, to a limited degree, across connectome weighting schemes, underscoring the peripheral regions' importance in maintaining and transitioning between diverse brain states.

### 3.7 Relationships of Regional Control Role and Other Node‐Centric Metrics

Our results show that high nodal degree regions in general, and the rich club in particular, contributed significantly little to optimal brain state control. In order to investigate which other features of cortical network organization might be relevant instead, we re‐examined possible associations of the regional rank measure with other candidate measures.

**FIGURE 7:**
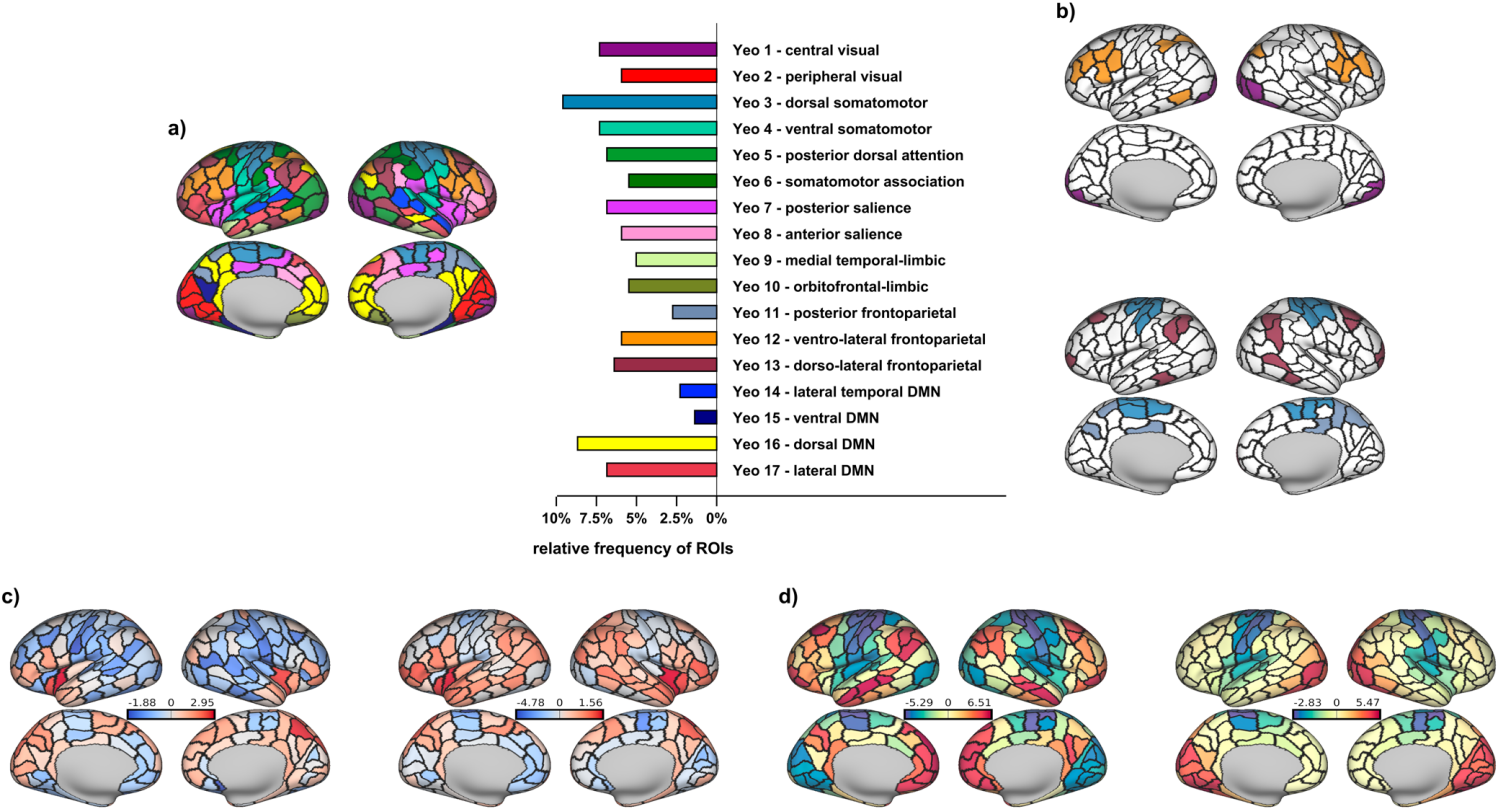
Who else, if not the rich club? Probing the role of intrinsic connectivity networks and cortical gradients in regional control. We systematically showed that the rich club does not optimally control brain state dynamics; but also that a different set of regions was task‐generally implicated as control nodes (see Figure 2). Here, we further characterized this set of top control nodes by comparing it to intrinsic connectivity networks (a and b) and probing its position on various cortical gradients (c and d). (a) Shows regional assignments to intrinsic connectivity networks as defined in the 17‐network solution in Yeo et al. (2011); regions of interest are as defined in the Lausanne subparcellation of the Desikan‐Killiany atlas. The bar chart shows the relative frequency with which a certain network was assigned to ROIs in our sample and includes network labels. (b) Regions in networks 1 and 12 contributed significantly more to regional control of brain dynamics than expected by chance (top); regions in networks 3, 11, and 13 (the latter only for stability measures) contributed significantly less than expected by chance (bottom). (c) The higher the degree (left; *z*‐scored mean nodal degree per region), the lower a region's contribution to control of brain state dynamics. This mirrors our results regarding the rich club. Interestingly, we found the same relationship for a possible antagonist to degree, the participation coefficient: the higher the participation coefficient, the lower a region's contribution to control (right; *z*‐scored mean participation coefficient per region). Both measures were highly correlated on a group level in our sample. (d) Regions higher on the visual‐sensorimotor (right) cortical gradient as defined in Margulies et al. (2016) contributed significantly to regional control. No significant relationship was found between regional control and regional position on the sensory‐transmodal cortical gradient (left). Warm colors indicate higher positions, cool colors lower positions on the respective gradient. DMN = default mode network.

We first defined a region's participation coefficient based on the canonical 17‐network‐division of the cerebral cortex (Yeo et al. 2011) and found that the higher a region's participation coefficient, the less it contributed to both the maintenance of and transitions between states (stability: r=0.18; p=0.0383, control energy: r=0.22; p=0.0164, spin test with 10,000 permutations). High nodal degree and high participation coefficient were thus both associated with decreased regional control contribution. Additionally, both measures correlated strongly with each other at the group level (r=0.54; p<0.001, see also Figure 7b).

We also investigated the significance of nodal communicability, and found that the higher a region's communicability, the less it contributed to control energy (r=0.25; p=0.0448, spin test with 10,000 permutations). The correlation between a region's communicability and its contribution to stability of a state however was nonsignificant (r=0.12; p=0.1943, spin test with 10,000 permutations).

Next, we examined the control role of various functional networks and their subdivisions (see Table S22 and Figure 7a). The primary visual network (Y1) and the ventrolateral division of the fronto‐parietal network (Y12) contributed significantly more to brain state stability and brain state transitions than expected by chance (all p<0.05, see Figure 7b, top). Conversely, specific subdivisions of three networks contributed less to control energy than expected by chance (all p<0.05), see Figure 7b, bottom: The dorsal division of the somato‐motor network (Y3) and the posterior division of the fronto‐parietal network (Y11) contributed less to both maintenance of states as well as transitions between states; while the dorso‐lateral division of the fronto‐parietal network (Y13) contributed less to maintenance of states only.

We also investigated the correlation between mean regional rank and that region's position on the sensory‐transmodal and visual‐sensorimotor cortical gradient (Margulies et al. 2016). A region's position on the visual‐sensorimotor, but not the sensory‐transmodal cortical gradient was significantly negatively associated with its regional contribution to both stability (r=−0.47; p<0.001) and control energy (r=−0.44; p<0.001; see also Figure 7d). This suggests that regions higher along the visual‐sensorimotor cortical gradient, that is, “more visual” regions, are more involved in control tasks.

Lastly, we explored possible relationships between mean rank of a region and how strongly structure and function are coupled in that region (Valk et al. 2022). We found no significant associations between mean rank regarding control contribution of a region and the extent of its structural‐functional coupling.

## 4 Discussion

The human brain's adaptability results from its ability to stabilize behaviorally appropriate activity patterns or to flexibly adjust its state to meet current demands (Benisty et al. 2024; Braun et al. 2015; Shine et al. 2016). While the brain uses the anatomy of its white matter scaffold for these adjustments (Cornblath et al. 2020; Honey et al. 2007; Sorrentino et al. 2021), the exact mechanisms steering these transitions are not fully understood.

One candidate for general brain state control is the rich club, a group of very highly interconnected hub regions (Gollo et al. 2015; Senden et al. 2017, 2018; Towlson et al. 2013; van den Heuvel et al. 2012).

We here used optimal network control and graph‐theoretical tools to investigate a possible control role of the rich club during maintenance of and transition between different brain states, constrained by seven canonical fMRI tasks. We found that rich club regions and high‐degree regions in general had consistently less impact on control metrics than peripheral regions. This finding was robust across different parameters and definitions, and only vanished partially when utilizing a binary connectome map, which is a highly simplified representation of the brain's anatomical scaffold that has been shown to disregard some biologically relevant detail (Bassett and Bullmore 2017; Wig et al. 2011).

Our findings challenge the traditional view that high‐degree regions—such as the rich club—constitute an integrative core essential for brain function control (Deco et al. 2017, 2021; de Pasquale et al. 2016; Zink et al. 2021) and efficient brain state transitions (Betzel et al. 2016). In contrast, when specifically examining control processes underlying cognitively relevant brain states, we did not observe a single brain state trajectory to which the rich club contributed more control energy than the reference set. Prior work suggests that the role of hub regions in network control may be neither universal nor state‐general, but instead depends on the ease with which a given state can be reached—implying that hubs are anatomically optimized for transitions to easily accessible states (Gu et al. 2015). These studies, however, focus on general transition capabilities rather than on concrete control processes for cognitively meaningful states. By applying optimal control analyses to such specific processes, we found no consistent evidence that transitions to more stable (i.e., energetically efficient) states require greater contributions from the rich club than transitions to less stable states. This suggests either that the brain states we examined are too difficult to reach for the rich club to exert effective control, or that the rich club plays a smaller role in state control dynamics than previously assumed. Notably, these findings hold true not only for brain states defined by task‐related activity patterns but also for those derived from resting‐state data.

Interestingly, regions with a high participation coefficient also did not emerge as top control nodes. The participation coefficient characterizes regions whose connections connect across module boundaries within the connectome. It has been argued that these diverse regions may be more involved than high‐degree rich club regions in the orchestration of information exchange in the brain network (Bertolero et al. 2017; Gollo et al. 2015; Lohia et al. 2024). However, the present data suggest that a putative “diverse club” defined on the basis of well‐known resting state networks and structural connections between them contributes as little to the control of brain state dynamics as the rich club and that indeed, increased cognitive effort might instead be associated more with diminishing importance of clubness and decreased modularity in the brain (Finc et al. 2017; Hearne et al. 2017; Kitzbichler et al. 2011).

Instead, a region's effectiveness in controlling brain state transitions may depend more on its membership in subdivisions of certain intrinsic connectivity networks or its position on specific cortical gradients of cortical functional connectivity. Specifically, regions within the primary visual network (Y1) and the ventrolateral division of the fronto‐parietal network (Y12) exhibited especially high task‐general control influences. Both the central visual and the ventrolateral frontoparietal resting state networks show clinically relevant alterations (Cieri et al. 2020): Changes in their dynamics have been associated with clinical status in disorders of consciousness (Heine et al. 2012; Medina et al. 2022), pain conditions (Shen et al. 2019; Sujanthan et al. 2022), psychiatric syndromes (Rai et al. 2021), and neurodegenerative disease (Canu et al. 2022; Filippi et al. 2019). Our findings demonstrate that the ventro‐lateral subdivision of the fronto‐parietal network exhibited significantly high task‐general control influences. This result is consistent with the dual network account of cognitive control, which identifies the fronto‐parietal network as one of two principal control networks (Cornblath et al. 2020; Dosenbach et al. 2007; Gu et al. 2015; Rosen et al. 2016; Seeley et al. 2007). According to this perspective, the fronto‐parietal network is implicated in domain‐general cognitive control and executive functioning (Schultz et al. 2022; Seeley et al. 2007), particularly over shorter timescales (Power and Petersen 2013). However, the dual network account presents a more nuanced view by differentiating between state transitions and state maintenance, attributing these functions to the fronto‐parietal and cingulo‐opercular networks, respectively (Dosenbach et al. 2007). Interestingly, our study revealed that the ventro‐lateral portion of the fronto‐parietal network was task‐generally involved in both the transitions between and maintenance of brain states. This suggests that the role of this subdivision in cognitive control may be more integrative than previously understood, encompassing both dynamic adjustments and sustained control processes. It is also noteworthy that in this present study, the posterior section, and for stability measures also the dorso‐lateral section, of the fronto‐parietal network had much like the rich club, a significantly negligible contribution to control of brain states. This finding underscores the heterogeneity within the fronto‐parietal network itself, challenging the idea that the entire fronto‐parietal network contributes to brain control. It thereby supports existing research stressing the biological, connectivity‐related and transcriptional distinctiveness of various fronto‐parietal subnetworks (Murphy et al. 2020).

The participation of the primary visual network (Y1) in control processes was also reflected in our findings regarding the sensory‐transmodal and visual‐sensorimotor cortical gradients: Being positioned higher on the visual‐sensorimotor gradient distinguishing somatomotor regions (lower) from visual regions (higher; Margulies et al. 2016) was significantly related to high task‐general control participation. The striate cortex might not only hold a special position as the starting point of the cortical hierarchy (Mesulam 1998), but also as a starting point for higher cognitive functions such as cognitive control (Gavornik and Bear 2014; Muckli 2010). Activity in the occipital cortex, particularly in the calcarine cortex and area V1, has been shown to precede cognitive control failures (Cieslik et al. 2024; Hamm et al. 2012; Su et al. 2020) and to encode multisensory integration (Murray et al. 2016). The visual network has also been shown to merge together with a frontoparietal module under increasing cognitive complexity of a task (Hearne et al. 2017). The classical account posits the primary visual network as a specific unisensory processing area, though this, also in light of our findings here, might constitute a significant oversimplification (Muckli 2010).

Methodological considerations need to be made: Following previous research (Braun et al. 2021) we use first‐level analysis results—specifically, spatial patterns of beta weights—as the basis for brain‐state definition. Therefore, brain states are resulting from statistical maps rather than direct BOLD‐level activity patterns. This anchors state definitions in behaviorally constrained task data, thus enhancing ecological validity, and extends earlier research that relied on simulated or resting state activity (Betzel et al. 2016; Gu et al. 2015, 2017). However, beta maps represent statistical patterns rather than instantaneous BOLD activity. As a consequence, they do not capture the rapid temporal fluctuations that shape spontaneous state exploration. Moreover, nodes that show similar levels of activation in both the initial and target states cannot be distinguished from nodes that are inactive in both conditions. This limits the ability to separate task‐general from task‐specific activity and may influence resulting control metrics. In addition, the linear framework used here offers a global view of controlled transitions but does not represent spontaneous or nonlinear reconfigurations of brain activity. Although large scale dynamics can often be approximated linearly at this resolution (Gu et al. 2015, 2017), we join others (Betzel et al. 2016; Ceballos et al. 2025; Parkes et al. 2024) in highlighting the need for nonlinear and time‐resolved extensions of NCT methodology to capture the full richness of ongoing neural dynamics. Importantly, we did not examine subcortical structures in detail, including thalamic nuclei and the basal ganglia loops known to support cognitive control (Aron et al. 2016). Their established role in cognitive control makes them an appealing target for future extensions of NCT. At the same time, cognitive control in the psychological sense differs from network control as formalized in NCT (Medaglia 2019), so the absence of these regions does not undermine the interpretation of our findings within this framework. Existing NCT work also suggests that subcortical areas contribute less strongly to optimal control than cortical regions (Braun et al. 2021), which indicates that their omission may have only limited influence on the present results. Combined with current methodological constraints, such as the lack of suitable spin test procedures for deep structures, these considerations motivated our decision to focus on cortical regions while highlighting subcortical NCT as an important direction for future research. Furthermore, the specific and circumscribed nature of the task data collected within the HCP should be considered when interpreting our results. Of note, while our results regarding the rich club held true for all task contrasts investigated here, we still found considerable differences between tasks, including much higher effect sizes when the target state was a language or motor condition. With network control theoretical approaches being a relatively recent approach in neuroscience, it is difficult to decipher whether this finding is based on methodological or cognitive properties of these two tasks. There are indications that the language and motor tasks are methodologically different from the other tasks employed in the HCP (possibly favorable signal‐to‐noise ratio and decreased interindividual variability; Rastegarnia et al. 2023). On the other hand, Luppi et al. (2024) investigate network control properties of meta‐analytically defined brain states and also find a language‐related target state to demand considerably high transition energy. Further research using much larger sets of brain states is needed to disentangle a possible methodological from a biologically grounded cause of task‐related differences in control metrics.

Given the ongoing debate about the precise interpretation of modal and average controllability metrics (Pasqualetti et al. 2019; Suweis et al. 2019; Tu et al. 2018), we employed a third methodological approach in our choice of optimal control analyses. While not directly focused on the rich club, previous optimal control analyses have shown that degree‐preserving null models perform worse than the empirical human connectome in control tasks—suggesting that nodal degree alone may not be the key determinant of effective control over brain state transitions (Luppi et al. 2024). Research by Betzel et al. (2016) has additionally suggested that rich club nodes might constitute optimal targets of control processes more than optimal controllers. Our findings confirm and extend these suggestions, offering that neither the rich club as a network, nor individual rich club nodes, seem suited toward optimally controlling the human connectome. Nonetheless, replicating our results using average and modal controllability metrics might prove fruitful. These two approaches allow for distinct investigations of the brain's general ability, irrespective of specific states, to control easily reachable and difficult to reach brain states (Karrer et al. 2020). Similar to Luppi et al. (2024) we found that the effect size of the control impact difference between the rich club and the reference set was partially related to target state characteristics. Other research has linked cognitive load to the specifics of state trajectories (Cornblath et al. 2020) and shown that cognitively effortful states are connected to increased control energetic effort (Braun et al. 2021; Luppi et al. 2024). Systematically considering target state specifics, such as associated cognitive load or reachability (Gu et al. 2015), can further elucidate network control processes and enhance approaches that respect current methodological boundaries of network control theoretical analyses (see also Karrer et al. 2020; Pasqualetti et al. 2019; Tu et al. 2018). Combined with the use of time‐resolved activity patterns, such as source‐reconstructed EEG or MEG data, this would allow for a closer study of the dynamics of control, including possibly regionally different timescales (Gollo et al. 2015).

## 5 Conclusion

We investigated the control‐theoretical role of the brain's rich club in the maintenance of cognitively relevant brain states, as well as in orchestrating transitions between states. Using HCP task‐fMRI data of seven canonical tasks, we were able to show that the rich club does not optimally control the stability of a state or transitions between states. Results instead indicate that peripheral regions, more specifically those affiliated with central‐visual and ventro‐lateral frontoparietal resting state networks, as well as those positioned higher on the visual‐sensorimotor cortical gradient, had a significantly higher impact on control metrics than the rich club. While these findings do not negate the importance or an integratory role of the rich club, they fit with an account describing it not as a control center, but instead a passive “data‐highway” of the human brain. Rich club regions may not actively drive network control, yet their repeatedly observed integratory importance can still arise from their high strength and high degree connections that form a strong network infrastructure, similar to how a highway enables efficient flow in transport networks.

## Author Contributions

A.N.P. contributed conceptualization, data curation, data analysis, writing – original draft, writing – review and editing. R.F.B. contributed resources, writing – review and editing, supervision. U.B. contributed resources, code, writing – review and editing. S.M. contributed conceptualization, resources, code, writing – review and editing, supervision.

## Funding

The authors have nothing to report.

## Conflicts of Interest

The authors declare no conflicts of interest.

## Supporting information


**Data S1:** hbm70485‐sup‐0001‐Supinfo1.pdf.

**Data S2:** hbm70485‐sup‐0002‐Supinfo2.pdf.

## Data Availability

The data that support the findings of this study are openly available in the Human Connectome Project at https://db.humanconnectome.org/.
