# Supplementary material for: Exploring the Role of the Rich Club in Network Control of Neurocognitive States
Source: Hum Brain Mapp. 2026 Feb 26;47(4):e70485. doi: 10.1002/hbm.70485 (PMC12945927; doi:10.1002/hbm.70485)
Supplement: Supplementary file 1 — Data S1: hbm70485‐sup‐0001‐Supinfo1.pdf. [file HBM-47-e70485-s002.pdf]

Table S1

Results of repeated measures ANOVA comparing global state stability of the instable and stable state

Measures	stability stable state		stability instable state		CV[a]	Main effect	
	M	*SD*	*M*	*SD*	*F* (1,96)	*F* (1,96)	η^2
Emotion Task	6.22e-6	2.13e-6	4.45e-6	1.42e-6	6.43*	43.04***	.31
Working Memory Task	5.31e-6	1.90e-6	4.47e-6	1.50e-6	0.04	29.62***	.24
Language Task	3.84e-6	1.37e-6	3.80e-6	1.51e-6	0.19	0.01	.00
Relational Task	3.91e-6	1.34e-6	3.53e-6	1.38e-6	7.72*	18.11***	.16
Social Task	5.83e-6	2.22e-6	4.55e-6	1.44e-6	0.67	44.23***	.32
Gambling Task	7.76e-6	3.15e-6	7.24e-6	2.84e-6	0.98	9.75**	.09
Motor Task	12.83e-6	4.71e-6	4.25e-6	1.85e-6	0.00	221.59***	.70

Note. This table demonstrates means and standard deviations of measures for stability of the stable as well as instable state, as well as corresponding test statistics of repeated measures ANOVA significance testing. Means and standard deviations are rounded to increase comparability for the reader.

[a] CV = covariate, here mean difference of activation between the instable and stable state

*** $p < 0.001$, ** $p < 0.01$, * $p < 0.05$

Table S2

Results of repeated measures ANOVA comparing global control energy transitioning between the stable and instable state

Measures	stable -> instable state		instable -> stable state		CV	Main effect	
	M	*SD*	*M*	*SD*	*F* (1,96)	*F* (1,96)	η^2
Emotion Task	4.21e+5	2.35e+5	2.37e+5	1.09e+5	25.56***	11.28**	.11
Working Memory Task	3.30e+5	1.24e+5	2.46e+5	1.04e+5	0.81	30.83***	.24
Language Task	4.97e+5	1.76e+5	4.78e+5	1.67e+5	0.72	0.00	.00
Relational Task	4.09e+5	1.78e+5	3.35e+5	2.09e+5	11.78***	26.63***	.22
Social Task	3.78e+5	1.77e+5	2.58e+5	1.74e+5	Jan 77	50.39***	.34
Gambling Task	2.09e+5	0.83e+5	1.87e+5	0.79e+5	0.03	5.57*	.05
Motor Task	5.96e+5	3.28e+5	1.53e+5	1.15e+5	8.64**	104.57***	.52

Note. This table demonstrates means and standard deviations of measures for control energy for the transition between the stable and instable state, as well as corresponding test statistics of repeated measures ANOVA significance testing. Means and standard deviations are rounded to increase comparability for the reader.

[a] CV = covariate, here mean difference of activation between the instable and stable state

*** $p < 0.001$, ** $p < 0.01$, * $p < 0.05$

Table S3

Results of repeated measures ANOVA comparing state stability between exclusion of rich club members and a size-matched set of semi-randomly selected regions

Measures	RC excluded		Random excluded		CV	Main effect	
	M	*SD*	*M*	*SD*	*F* (1,96)	*F* (1,96)	η^2
Stability stable state							
Emotion Task	5.74E-08	2.89E-09	4.23E-08	1.57E-09	0.02	15.62***	.14
Working Memory Task	4.76E-08	2.72E-09	3.75E-08	1.46E-09	0.11	12.19***	.12
Language Task	4.47E-08	2.73E-09	2.29E-08	8.41E-10	0.67	30.84***	.24
Relational Task	3.24E-08	1.49E-09	2.74E-08	1.01E-09	0.05	9.96**	.09
Social Task	5.00E-08	2.61E-09	4.10E-08	1.74E-09	0.07	10.43**	.10
Gambling Task	7.57E-08	3.84E-09	5.32E-08	2.48E-09	0.74	16.15***	.14
Motor Task	1.83E-07	1.11E-08	8.29E-08	3.38E-09	0.23	62.94***	.40
Stability instable state							
Emotion Task	4.81E-08	2.40E-09	2.96E-08	1.01E-09	0.24	38.30***	.29
Working Memory Task	3.78E-08	1.92E-09	3.08E-08	1.14E-09	0.12	14.11***	.13
Language Task	4.22E-08	2.36E-09	2.25E-08	8.91E-10	0.27	36.66***	.28
Relational Task	2.82E-08	1.60E-09	2.48E-08	1.01E-09	0.09	5.82*	.06
Social Task	3.99E-08	1.94E-09	3.09E-08	1.11E-09	0.02	16.91***	.15
Gambling Task	7.20E-08	4.00E-09	5.02E-08	2.26E-09	0.38	15.82***	.14
Motor Task	6.66E-08	4.81E-09	2.28E-08	1.08E-09	7.24**	86.50***	.47

Note. This table demonstrates means and standard deviations of measures for stability of the instable and stable state, as well as corresponding test statistics of repeated measures ANOVA significance testing. Means and standard deviations are rounded to increase comparability for the reader.

[a] CV = covariate, here mean difference of activation between the instable and stable state

*** $p < 0.001$, ** $p < 0.01$, * $p < 0.05$

Table S4

Results of repeated measures ANOVA comparing control energy between exclusion of rich club members and a size-matched set of semi-randomly selected regions

Measures	RC excluded		Random excluded		CV	Main effect	
	M	*SD*	*M*	*SD*	*F* (1,96)	*F* (1,96)	η^2
Energy stable -> instable							
Emotion Task	3.26E+07	1.83E+06	5.95E+07	3.34E+06	8.27**	30.05***	.24
Working Memory Task	3.98E+07	1.72E+06	4.69E+07	1.79E+06	0.88	5.14*	.05
Language Task	4.45E+07	2.50E+06	7.50E+07	2.65E+06	1.62	55.77***	.37
Relational Task	5.07E+07	2.24E+06	5.56E+07	2.61E+06	0.00	2.26	.02
Social Task	4.06E+07	2.19E+06	5.36E+07	2.42E+06	7.23**	12.41***	.11
Gambling Task	2.14E+07	1.16E+06	2.90E+07	1.15E+06	1.31	21.86***	.19
Motor Task	4.22E+07	3.51E+06	1.10E+08	5.63E+06	0.28	122.12***	.56
Energy instable -> stable							
Emotion Task	2.47E+07	1.53E+06	3.26E+07	1.45E+06	0.30	14.79***	.13
Working Memory Task	2.81E+07	1.55E+06	3.34E+07	1.39E+06	2.22	19.92***	.17
Language Task	4.11E+07	2.16E+06	7.21E+07	2.52E+06	3.94*	89.31***	.48
Relational Task	3.87E+07	2.58E+06	4.62E+07	2.84E+06	1.14	4.10*	.04
Social Task	2.99E+07	1.87E+06	3.45E+07	1.91E+06	0.81	9.62**	.09
Gambling Task	1.93E+07	1.06E+06	2.64E+07	1.11E+06	4.19*	15.91***	.14
Motor Task	9.43E+06	8.32E+05	2.18E+07	1.21E+06	15.01***	123.55***	.56

Note. This table demonstrates means and standard deviations of measures for control energy for the transition between the stable and instable state, as well as corresponding test statistics of repeated measures ANOVA significance testing. Means and standard deviations are rounded to increase comparability for the reader.

[a] CV = covariate, here mean difference of activation between the stable and instable state

*** p < 0.001, **p < 0.01, *p < 0.05

Table S5

Results of repeated measures ANOVA comparing stability of the stable state between exclusion of rich club members and a size-matched set of semi-randomly selected regions for various settings of the time horizon parameter T

From:	RC excluded		Random excluded		CV	Main effect	
	M	*SD*	*M*	*SD*	*F* (1,96)	*F* (1,96)	η^2
Emotion Task							
T = 0.5	1.56E-08	7.91E-10	1.15E-08	4.18E-10	0.00	15.70***	.14
T = 1.5	1.14E-07	5.71E-09	8.43E-08	3.05E-09	0.00	15.47***	.14
T = 2	1.74E-07	8.70E-09	1.29E-07	4.68E-09	0.01	15.32***	.14
T = 2.5	2.32E-07	1.16E-08	1.72E-07	6.24E-09	0.01	15.20***	.14
T = 3	2.85E-07	1.42E-08	2.12E-07	7.67E-09	0.01	15.14***	.14
Working Memory Task							
T = 0.5	1.30E-08	7.42E-10	1.02E-08	3.93E-10	0.10	12.19***	.11
T = 1.5	9.44E-08	5.38E-09	7.44E-08	2.87E-09	0.10	11.96***	.11
T = 2	1.44E-07	8.21E-09	1.14E-07	4.40E-09	0.10	11.79***	.11
T = 2.5	1.92E-07	1.09E-08	1.52E-07	5.86E-09	0.10	11.66***	.11
T = 3	2.35E-07	1.34E-08	1.87E-07	7.20E-09	0.10	11.57***	.11
Language Task							
T = 0.5	1.22E-08	7.48E-10	6.22E-09	2.32E-10	0.25	30.92***	.24
T = 1.5	8.84E-08	5.38E-09	4.55E-08	1.70E-09	0.26	30.84***	.24
T = 2	1.35E-07	8.20E-09	6.98E-08	2.61E-09	0.27	30.70***	.24
T = 2.5	1.79E-07	1.09E-08	9.32E-08	3.48E-09	0.28	30.55***	.24
T = 3	2.20E-07	1.33E-08	1.15E-07	4.28E-09	0.28	30.45***	.24
Relational Task							
T = 0.5	8.84E-09	4.09E-10	7.50E-09	2.70E-10	0.13	9.87**	.09
T = 1.5	6.43E-08	2.95E-09	5.49E-08	1.98E-09	0.14	9.49**	.09
T = 2	9.82E-08	4.48E-09	8.42E-08	3.03E-09	0.14	9.24**	.09
T = 2.5	1.31E-07	5.95E-09	1.12E-07	4.04E-09	0.14	9.03**	.09
T = 3	1.60E-07	7.28E-09	1.38E-07	4.97E-09	0.15	8.90**	.09
Social Task							
T = 0.5	1.36E-08	7.13E-10	1.12E-08	4.73E-10	0.00	11.66***	.11
T = 1.5	9.92E-08	5.18E-09	8.19E-08	3.45E-09	0.00	11.36**	.11
T = 2	1.52E-07	7.92E-09	1.26E-07	5.28E-09	0.00	11.18**	.10
T = 2.5	2.02E-07	1.05E-08	1.67E-07	7.03E-09	0.00	11.04**	.10
T = 3	2.48E-07	1.29E-08	2.06E-07	8.65E-09	0.00	10.98**	.10
Gambling Task							
T = 0.5	2.07E-08	1.05E-09	1.46E-08	7.05E-10	0.94	13.06***	.12
T = 1.5	1.50E-07	7.55E-09	1.06E-07	5.15E-09	0.97	12.91***	.12
T = 2	2.29E-07	1.15E-08	1.63E-07	7.88E-09	0.98	12.79***	.12
T = 2.5	3.04E-07	1.52E-08	2.18E-07	1.05E-08	0.99	12.70***	.12
T = 3	3.73E-07	1.86E-08	2.68E-07	1.29E-08	0.99	12.64***	.12
Motor Task							
T = 0.5	4.99E-08	3.04E-09	2.27E-08	9.47E-10	0.16	59.49***	.38
T = 1.5	3.62E-07	2.17E-08	1.66E-07	6.92E-09	0.14	60.75***	.39
T = 2	5.53E-07	3.30E-08	2.55E-07	1.06E-08	0.13	61.27***	.39
T = 2.5	7.36E-07	4.38E-08	3.40E-07	1.42E-08	0.12	61.63***	.39
T = 3	9.05E-07	5.36E-08	4.18E-07	1.74E-08	0.11	61.88***	.39

Note. This table demonstrates means and standard deviations of measures for stability of the stable state, as well as corresponding test statistics of repeated measures ANOVA significance testing. Means and standard deviations are rounded to increase comparability for the reader.

[a] CV = covariate, here mean difference of activation between the instable and stable state

*** $p < 0.001$, ** $p < 0.01$, * $p < 0.05$

Table S6

Results of repeated measures ANOVA comparing stability of the instable state between exclusion of rich club members and a size-matched set of semi-randomly selected regions for various settings of the time horizon parameter T

From:	RC excluded		Random excluded		CV	Main effect	
	M	*SD*	*M*	*SD*	*F* (1,96)	*F* (1,96)	η^2
Emotion Task							
T = 0.5	1.31E-08	6.58E-10	8.12E-09	2.80E-10	0.17	36.84***	.28
T = 1.5	9.52E-08	4.74E-09	5.93E-08	2.05E-09	0.16	36.47***	.28
T = 2	1.45E-07	7.23E-09	9.10E-08	3.14E-09	0.15	36.19***	.27
T = 2.5	1.93E-07	9.60E-09	1.21E-07	4.19E-09	0.15	35.95***	.27
T = 3	2.37E-07	1.18E-08	1.49E-07	5.15E-09	0.15	35.81***	.27
Working Memory Task							
T = 0.5	1.03E-08	5.25E-10	8.41E-09	3.08E-10	0.08	12.70***	.12
T = 1.5	7.49E-08	3.78E-09	6.14E-08	2.25E-09	0.09	12.41***	.11
T = 2	1.14E-07	5.76E-09	9.41E-08	3.45E-09	0.10	12.23***	.11
T = 2.5	1.52E-07	7.63E-09	1.25E-07	4.60E-09	0.11	12.09***	.11
T = 3	1.86E-07	9.34E-09	1.54E-07	5.65E-09	0.11	12.02***	.11
Language Task							
T = 0.5	1.15E-08	6.48E-10	6.12E-09	2.63E-10	0.31	37.84***	.28
T = 1.5	8.36E-08	4.66E-09	4.48E-08	1.93E-09	0.37	37.61***	.28
T = 2	1.28E-07	7.10E-09	6.88E-08	2.97E-09	0.41	37.33***	.28
T = 2.5	1.70E-07	9.42E-09	9.18E-08	3.96E-09	0.45	37.06***	.28
T = 3	2.09E-07	1.15E-08	1.13E-07	4.88E-09	0.48	36.87***	.28
Relational Task							
T = 0.5	7.69E-09	4.36E-10	6.81E-09	2.76E-10	0.11	5.11*	.05
T = 1.5	5.58E-08	3.15E-09	4.98E-08	2.01E-09	0.13	4.82*	.05
T = 2	8.53E-08	4.81E-09	7.63E-08	3.08E-09	0.14	4.64*	.05
T = 2.5	1.13E-07	6.39E-09	1.02E-07	4.11E-09	0.15	4.49*	.04
T = 3	1.39E-07	7.84E-09	1.25E-07	5.05E-09	0.15	4.40*	.04
Social Task							
T = 0.5	1.09E-08	5.30E-10	8.45E-09	3.06E-10	0.01	16.51***	.15
T = 1.5	7.91E-08	3.83E-09	6.16E-08	2.23E-09	0.01	16.25***	.14
T = 2	1.21E-07	5.83E-09	9.44E-08	3.41E-09	0.01	16.07***	.14
T = 2.5	1.61E-07	7.73E-09	1.26E-07	4.54E-09	0.01	15.94***	.14
T = 3	1.97E-07	9.48E-09	1.55E-07	5.58E-09	0.02	15.89***	.14
Gambling Task							
T = 0.5	1.97E-08	1.10E-09	1.36E-08	6.15E-10	0.68	14.53***	.13
T = 1.5	1.42E-07	7.86E-09	9.97E-08	4.49E-09	0.69	14.50***	.13
T = 2	2.17E-07	1.19E-08	1.53E-07	6.89E-09	0.69	14.43***	.13
T = 2.5	2.88E-07	1.57E-08	2.04E-07	9.19E-09	0.69	14.35***	.13
T = 3	3.54E-07	1.92E-08	2.51E-07	1.13E-08	0.70	14.31***	.13
Motor Task							
T = 0.5	1.82E-08	1.33E-09	6.30E-09	3.15E-10	7.03**	84.72***	.47
T = 1.5	1.32E-07	9.41E-09	4.62E-08	2.31E-09	7.10**	87.99***	.48
T = 2	2.01E-07	1.42E-08	7.08E-08	3.55E-09	7.11**	89.47***	.48
T = 2.5	2.68E-07	1.88E-08	9.45E-08	4.74E-09	7.11**	90.59***	.49
T = 3	3.29E-07	2.30E-08	1.16E-07	5.83E-09	7.11**	91.46***	.49

Note. This table demonstrates means and standard deviations of measures for stability of the instable state, as well as corresponding test statistics of repeated measures ANOVA significance testing. Means and standard deviations are rounded to increase comparability for the reader.

[a] CV = covariate, here mean difference of activation between the instable and stable state

*** $p < 0.001$, ** $p < 0.01$, * $p < 0.05$

Table S7

Results of repeated measures ANOVA comparing control energy when traversing from the stable to the instable state for various settings of the time horizon parameter T; comparing exclusion of rich club members and a size-matched set of semi-randomly selected regions

From:	RC excluded		Random excluded		CV	Main effect	
	M	*SD*	*M*	*SD*	*F* (1,96)	*F* (1,96)	η^2
Emotion Task							
T = 0.5	1.84E+08	1.22E+07	4.07E+08	2.45E+07	16.21***	38.57***	.29
T = 1.5	1.46E+07	7.78E+05	2.41E+07	1.29E+06	5.50*	26.49***	.22
T = 2	9.10E+06	4.75E+05	1.43E+07	7.52E+05	4.05*	24.82***	.21
T = 2.5	6.68E+06	3.46E+05	1.03E+07	5.32E+05	3.28	23.92***	.20
T = 3	5.37E+06	2.77E+05	8.13E+06	4.19E+05	2.84	23.45***	.20
Working Memory Task							
T = 0.5	2.14E+08	9.84E+06	2.68E+08	1.20E+07	4.02*	5.67*	.06
T = 1.5	1.80E+07	7.83E+05	2.06E+07	7.29E+05	0.07	4.95*	.05
T = 2	1.13E+07	4.95E+05	1.27E+07	4.47E+05	0.00	5.01*	.05
T = 2.5	8.30E+06	3.66E+05	9.32E+06	3.25E+05	0.02	5.04*	.05
T = 3	6.68E+06	2.96E+05	7.48E+06	2.61E+05	0.05	5.08*	.05
Language Task							
T = 0.5	3.15E+08	1.77E+07	5.68E+08	2.00E+07	3.82	61.60***	.39
T = 1.5	1.85E+07	1.06E+06	3.08E+07	1.11E+06	1.74	56.09***	.37
T = 2	1.12E+07	6.54E+05	1.84E+07	6.68E+05	1.52	54.38***	.36
T = 2.5	8.13E+06	4.77E+05	1.32E+07	4.83E+05	1.42	53.29***	.36
T = 3	6.49E+06	3.83E+05	1.05E+07	3.85E+05	1.37	52.64***	.35
Relational Task							
T = 0.5	2.54E+08	1.28E+07	2.89E+08	1.41E+07	2.94	1.02	.01
T = 1.5	2.35E+07	1.01E+06	2.52E+07	1.18E+06	0.26	2.28	.02
T = 2	1.49E+07	6.39E+05	1.59E+07	7.43E+05	0.46	2.31	.02
T = 2.5	1.10E+07	4.74E+05	1.17E+07	5.49E+05	0.57	2.27	.02
T = 3	8.90E+06	3.83E+05	9.42E+06	4.43E+05	0.64	2.24	.02
Social Task							
T = 0.5	2.48E+08	1.55E+07	3.62E+08	2.13E+07	21.51***	9.27**	.09
T = 1.5	1.77E+07	9.22E+05	2.24E+07	9.69E+05	3.21	9.00**	.09
T = 2	1.09E+07	5.62E+05	1.35E+07	5.70E+05	1.81	8.48**	.08
T = 2.5	7.95E+06	4.09E+05	9.70E+06	4.07E+05	1.24	8.07**	.08
T = 3	6.38E+06	3.28E+05	7.72E+06	3.23E+05	0.96	7.82**	.08
Gambling Task							
T = 0.5	1.17E+08	7.55E+06	1.71E+08	8.23E+06	4.94*	15.94***	.14
T = 1.5	9.77E+06	5.08E+05	1.29E+07	4.92E+05	0.22	23.54***	.20
T = 2	6.15E+06	3.15E+05	8.03E+06	3.04E+05	0.07	24.00***	.20
T = 2.5	4.55E+06	2.31E+05	5.89E+06	2.23E+05	0.03	24.04***	.20
T = 3	3.67E+06	1.86E+05	4.74E+06	1.79E+05	0.01	24.02***	.20
Motor Task							
T = 0.5	3.36E+08	3.07E+07	9.18E+08	5.09E+07	0.00	117.76***	.55
T = 1.5	1.58E+07	1.24E+06	3.94E+07	2.04E+06	0.35	116.36***	.55
T = 2	8.98E+06	6.82E+05	2.19E+07	1.11E+06	0.44	115.63***	.55
T = 2.5	6.26E+06	4.65E+05	1.50E+07	7.58E+05	0.49	114.95***	.54
T = 3	4.88E+06	3.58E+05	1.16E+07	5.83E+05	0.51	114.40***	.54

Note. This table demonstrates means and standard deviations of measures for energy needed for transitions from the stable to the instable state, as well as corresponding test statistics of repeated measures ANOVA significance testing. Means and standard deviations are rounded to increase comparability for the reader.

[a] CV = covariate, here mean difference of activation between the instable and stable state

*** $p < 0.001$, ** $p < 0.01$, * $p < 0.05$

Table S8

Results of repeated measures ANOVA comparing control energy when traversing from the instable to the stable state for various settings of the time horizon parameter T; comparing exclusion of rich club members and a size-matched set of semi-randomly selected regions

From:		RC excluded		Random excluded		CV	Main effect	
		M	*SD*	*M*	*SD*	*F* (1,96)	*F* (1,96)	η^2
Emotion Task								
	T = 0.5	1.30E+08	8.80E+06	2.23E+08	1.06E+07	5.49*	30.92***	.24
	T = 1.5	1.16E+07	6.88E+05	1.45E+07	6.51E+05	0.02	13.30***	.12
	T = 2	7.45E+06	4.31E+05	9.15E+06	4.11E+05	0.05	12.73***	.11
	T = 2.5	5.55E+06	3.18E+05	6.80E+06	3.06E+05	0.05	12.53***	.12
	T = 3	4.51E+06	2.56E+05	5.51E+06	2.48E+05	0.05	12.46***	.11
Working Memory Task								
	T = 0.5	1.33E+08	8.15E+06	1.78E+08	8.47E+06	0.37	31.26***	.25
	T = 1.5	1.37E+07	7.29E+05	1.58E+07	6.11E+05	3.41	16.50***	.15
	T = 2	8.95E+06	4.67E+05	1.02E+07	3.87E+05	3.38	14.31***	.13
	T = 2.5	6.73E+06	3.48E+05	7.58E+06	2.87E+05	3.34	13.18***	.12
	T = 3	5.48E+06	2.82E+05	6.16E+06	2.32E+05	3.32	12.58***	.12
Language Task								
	T = 0.5	2.92E+08	1.26E+07	5.42E+08	1.76E+07	7.27**	90.78***	.49
	T = 1.5	1.72E+07	9.88E+05	2.95E+07	1.06E+06	3.11	70.89***	.42
	T = 2	1.05E+07	6.21E+05	1.77E+07	6.50E+05	2.63	66.64***	.41
	T = 2.5	7.62E+06	4.57E+05	1.28E+07	4.73E+05	2.41	64.55***	.40
	T = 3	6.09E+06	3.68E+05	1.02E+07	3.79E+05	2.31	63.52***	.40
Relational Task								
	T = 0.5	1.72E+08	1.49E+07	2.26E+08	1.77E+07	14.77***	2.91	.03
	T = 1.5	1.91E+07	1.15E+06	2.19E+07	1.24E+06	0.02	4.17*	.04
	T = 2	1.25E+07	7.16E+05	1.41E+07	7.68E+05	0.04	4.14*	.04
	T = 2.5	9.37E+06	5.27E+05	1.05E+07	5.62E+05	0.15	4.09*	.04
	T = 3	7.64E+06	4.25E+05	8.50E+06	4.52E+05	0.24	4.07*	.04
Social Task								
	T = 0.5	1.75E+08	1.43E+07	2.29E+08	1.87E+07	8.56**	7.03**	.07
	T = 1.5	1.37E+07	7.76E+05	1.54E+07	7.46E+05	1.19	10.14**	.10
	T = 2	8.70E+06	4.75E+05	9.71E+06	4.44E+05	1.96	10.33**	.10
	T = 2.5	6.46E+06	3.47E+05	7.19E+06	3.21E+05	2.30	10.39**	.10
	T = 3	5.23E+06	2.78E+05	5.81E+06	2.56E+05	2.46	10.47**	.10
Gambling Task								
	T = 0.5	1.02E+08	6.34E+06	1.52E+08	8.07E+06	13.18***	10.60**	.10
	T = 1.5	8.98E+06	4.82E+05	1.19E+07	4.86E+05	1.70	17.04***	.15
	T = 2	5.72E+06	3.03E+05	7.49E+06	3.01E+05	0.99	17.98***	.16
	T = 2.5	4.25E+06	2.24E+05	5.53E+06	2.21E+05	0.70	18.37***	.16
	T = 3	3.44E+06	1.81E+05	4.46E+06	1.78E+05	0.55	18.58***	.16
Motor Task								
	T = 0.5	1.10E+08	1.22E+07	3.12E+08	1.89E+07	9.58**	130.45***	.58
	T = 1.5	3.72E+06	2.58E+05	7.57E+06	3.95E+05	8.48**	92.63***	.49
	T = 2	2.34E+06	1.44E+05	4.57E+06	2.25E+05	3.54	93.53***	.49
	T = 2.5	1.76E+06	1.02E+05	3.40E+06	1.62E+05	1.75	97.86***	.50
	T = 3	1.44E+06	8.18E+04	2.78E+06	1.30E+05	1.05	101.39***	.51

Note. This table demonstrates means and standard deviations of measures for energy needed for transitions from the instable to the stable state, as well as corresponding test statistics of repeated measures ANOVA significance testing. Means and standard deviations are rounded to increase comparability for the reader.

[a] CV = covariate, here mean difference of activation between the instable and stable state

*** p < 0.001, ** p < 0.01, * p < 0.05

Table S9

Results of repeated measures ANOVA comparing state stability between exclusion of rich club members and a size-matched set of semi-randomly selected regions; analyses based on a NOS-weighted connectome; outlier not included

Measures		RC excluded		Random excluded		CV	Main effect	
		M	*SD*	*M*	*SD*	*F* (1,96)	*F* (1,96)	η^2
Stability stable state								
	Emotion Task	6.30E-08	4.70E-09	2.28E-08	1.45E-09	0.07	42.40***	.31
	Working Memory Task	6.65E-08	5.99E-09	2.67E-08	1.90E-09	3.75	43.96***	.32
	Language Task	3.87E-08	4.03E-09	1.26E-08	7.93E-10	0.01	18.86***	.17
	Relational Task	5.21E-08	3.92E-09	1.88E-08	1.33E-09	0.03	46.25***	.33
	Social Task	6.29E-08	4.97E-09	3.25E-08	2.36E-09	0.44	36.73***	.28
	Gambling Task	8.99E-08	6.34E-09	3.39E-08	2.64E-09	0.13	48.08***	.34
	Motor Task	1.90E-07	1.85E-08	4.32E-08	3.13E-09	0.41	42.05***	.31
Stability instable state								
	Emotion Task	5.44E-08	4.37E-09	1.72E-08	1.11E-09	0.06	40.21***	.30
	Working Memory Task	5.25E-08	4.34E-09	2.17E-08	1.62E-09	0.50	41.21***	.30
	Language Task	3.39E-08	3.03E-09	1.36E-08	8.94E-10	0.02	19.91***	.17
	Relational Task	4.29E-08	3.34E-09	1.72E-08	1.33E-09	0.31	50.25***	.35
	Social Task	5.23E-08	4.25E-09	2.44E-08	1.68E-09	1.10	38.02***	.29
	Gambling Task	8.73E-08	6.58E-09	3.25E-08	2.29E-09	0.00	37.81***	.28
	Motor Task	6.19E-08	5.00E-09	1.18E-08	1.06E-09	2.40	75.24***	.44

Note. This table demonstrates means and standard deviations of measures for stability of the stable and instable state, as well as corresponding test statistics of repeated measures ANOVA significance testing. Means and standard deviations are rounded to increase comparability for the reader. **Note that one participant was identified as an outlier (energy values > 2IQR distance from the group) and subsequently excluded from inferential analyses. See Supplemental Figure S4 and Supplemental Tables S11 and S12 for a visualization of the outlier.**

[a] CV = covariate, here mean difference of activation between the stable and instable state

*** $p < 0.001$, ** $p < 0.01$, * $p < 0.05$

Table S10

Results of repeated measures ANOVA comparing control energy between exclusion of rich club members and a size-matched set of semi-randomly selected regions; analyses based on a NOS-weighted connectome; outlier not included

Measures		RC excluded		Random excluded		CV	Main effect	
		M	*SD*	*M*	*SD*	*F* (1,96)	*F* (1,96)	η^2
Energy stable -> instable								
	Emotion Task	4.81E+07	4.77E+06	1.50E+08	1.69E+07	8.81**	6.23*	.06
	Working Memory Task	4.43E+07	3.98E+06	1.03E+08	1.07E+07	1.95	6.81*	.07
	Language Task	8.92E+07	9.60E+06	1.74E+08	1.84E+07	0.39	5.94*	.06
	Relational Task	5.08E+07	4.49E+06	1.18E+08	1.03E+07	1.85	15.60***	.14
	Social Task	4.96E+07	4.68E+06	9.80E+07	7.87E+06	3.50	12.12***	.11
	Gambling Task	2.56E+07	2.28E+06	6.79E+07	6.84E+06	0.23	19.77***	.17
	Motor Task	6.38E+07	6.72E+06	3.41E+08	3.42E+07	0.09	36.81***	.28
Energy instable -> stable								
	Emotion Task	3.20E+07	3.40E+06	8.49E+07	8.56E+06	0.08	15.64***	.14
	Working Memory Task	3.49E+07	4.65E+06	6.89E+07	5.68E+06	0.06	15.19***	.14
	Language Task	7.41E+07	9.64E+06	1.80E+08	1.52E+07	0.02	19.21***	.17
	Relational Task	4.04E+07	5.22E+06	9.72E+07	9.10E+06	12.84***	11.61***	.11
	Social Task	3.78E+07	4.41E+06	6.64E+07	6.39E+06	3.77	11.80***	.11
	Gambling Task	2.50E+07	2.47E+06	6.46E+07	6.21E+06	0.35	22.82***	.19
	Motor Task	1.44E+07	1.84E+06	5.70E+07	4.65E+06	6.17*	29.18***	.24

Note. This table demonstrates means and standard deviations of measures for control energy for the transition between the stable and instable state, as well as corresponding test statistics of repeated measures ANOVA significance testing. Means and standard deviations are rounded to increase comparability for the reader. **Note that one participant was identified as an outlier (energy values > 2IQR distance from the group) and subsequently excluded from inferential analyses. See figure Supplemental Figure S4 and Supplemental Tables S11 and S12 for a visualization of the outlier.**

[a] CV = covariate, here mean difference of activation between the stable and instable state

*** p < 0.001, **p < 0.01, *p < 0.05

Table S11

Results of repeated measures ANOVA comparing state stability between exclusion of rich club members and a size-matched set of semi-randomly selected regions; analyses based on a NOS-weighted connectome; all 98 participants included

Measures	RC excluded		Random excluded		CV	Main effect	
	M	*SD*	*M*	*SD*	*F* (1,96)	*F* (1,96)	η^2
Stability stable state							
Emotion Task	6.25E-08	4.67E-09	2.26E-08	1.45E-09	0.07	42.47***	.31
Working Memory Task	6.64E-08	5.93E-09	2.64E-08	1.89E-09	3.81	44.97***	.32
Language Task	3.87E-08	3.99E-09	1.25E-08	7.95E-10	0.00	19.11***	.17
Relational Task	5.20E-08	3.88E-09	1.86E-08	1.33E-09	0.03	47.49***	.33
Social Task	6.30E-08	4.92E-09	3.22E-08	2.35E-09	0.42	37.71***	.28
Gambling Task	9.06E-08	6.31E-09	3.37E-08	2.62E-09	0.13	48.99***	.33
Motor Task	1.90E-07	1.83E-08	4.27E-08	3.13E-09	0.43	43.18***	.31
Stability instable state							
Emotion Task	5.41E-08	4.34E-09	1.71E-08	1.11E-09	0.06	40.48***	.30
Working Memory Task	5.24E-08	4.30E-09	2.15E-08	1.62E-09	0.53	42.40***	.31
Language Task	3.40E-08	3.00E-09	1.35E-08	8.91E-10	0.03	20.15***	.17
Relational Task	4.28E-08	3.30E-09	1.70E-08	1.33E-09	0.34	51.89***	.35
Social Task	5.23E-08	4.20E-09	2.42E-08	1.68E-09	1.09	38.92***	.29
Gambling Task	8.78E-08	6.54E-09	3.22E-08	2.28E-09	0.00	38.77***	.29
Motor Task	6.19E-08	4.94E-09	1.17E-08	1.05E-09	2.43	76.88***	.44

Note. This table demonstrates means and standard deviations of measures for stability of the stable and instable state, as well as corresponding test statistics of repeated measures ANOVA significance testing. Means and standard deviations are rounded to increase comparability for the reader.

[a] CV = covariate, here mean difference of activation between the stable and instable state

*** $p < 0.001$, ** $p < 0.01$, * $p < 0.05$

Table S12

Results of repeated measures ANOVA comparing control energy between exclusion of rich club members and a size-matched set of semi-randomly selected regions; analyses based on a NOS-weighted connectome; all 98 participants included

Measures	RC excluded		Random excluded		CV	Main effect	
	M	*SD*	*M*	*SD*	*F* (1,96)	*F* (1,96)	η^2
Energy stable -> instable							
Emotion Task	4.80E+07	4.72E+06	1.94E+08	4.72E+07	0.75	2.07	.02
Working Memory Task	4.42E+07	3.95E+06	1.10E+08	1.26E+07	0.89	7.41**	.07
Language Task	8.85E+07	9.53E+06	1.75E+08	1.82E+07	0.46	6.05*	.06
Relational Task	5.06E+07	4.45E+06	1.98E+08	8.04E+07	0.14	2.35	.02
Social Task	4.93E+07	4.64E+06	9.85E+07	7.81E+06	3.54	12.64***	.12
Gambling Task	2.54E+07	2.26E+06	6.87E+07	6.82E+06	0.23	20.49***	.18
Motor Task	6.34E+07	6.65E+06	4.11E+08	7.80E+07	0.06	10.97**	.10
Energy instable -> stable							
Emotion Task	3.22E+07	3.38E+06	9.57E+07	1.37E+07	0.02	9.17**	.09
Working Memory Task	3.47E+07	4.61E+06	7.16E+07	6.23E+06	0.15	15.71***	.14
Language Task	7.37E+07	9.55E+06	2.34E+08	5.58E+07	0.22	1.96	.02
Relational Task	4.03E+07	5.16E+06	1.10E+08	1.55E+07	1.34	6.35*	.06
Social Task	3.75E+07	4.37E+06	7.19E+07	8.33E+06	1.62	6.89*	.07
Gambling Task	2.48E+07	2.45E+06	6.51E+07	6.17E+06	0.35	23.58***	.20
Motor Task	1.43E+07	1.82E+06	7.62E+07	1.97E+07	0.15	3.72	.04

Note. This table demonstrates means and standard deviations of measures for control energy for the transition between the stable and instable state, as well as corresponding test statistics of repeated measures ANOVA significance testing. Means and standard deviations are rounded to increase comparability for the reader.

[a] CV = covariate, here mean difference of activation between the stable and instable state

*** $p < 0.001$, ** $p < 0.01$, * $p < 0.05$

Table S13

Results of repeated measures ANOVA comparing state stability between exclusion of rich club members and a size-matched set of semi-randomly selected regions; analyses based on a binary connectome

Measures	RC excluded		Random excluded		CV	Main effect	
	M	*SD*	*M*	*SD*	*F* (1,96)	*F* (1,96)	η^2
Stability stable state							
Emotion Task	5.79E-08	2.99E-09	5.25E-08	1.91E-09	0.20	1.16	.01
Working Memory Task	4.65E-08	2.56E-09	4.76E-08	1.82E-09	0.04	0.06	.00
Language Task	4.79E-08	2.80E-09	3.03E-08	1.09E-09	0.65	18.52***	.16
Relational Task	3.31E-08	1.54E-09	3.47E-08	1.26E-09	0.01	1.10	.01
Social Task	5.03E-08	2.66E-09	5.05E-08	2.10E-09	0.00	0.00	.00
Gambling Task	7.58E-08	3.96E-09	6.63E-08	2.91E-09	0.16	2.45	.02
Motor Task	2.01E-07	1.29E-08	1.04E-07	4.18E-09	0.02	44.94***	.32
Stability instable state							
Emotion Task	4.88E-08	2.44E-09	3.89E-08	1.30E-09	0.01	9.95**	.09
Working Memory Task	3.76E-08	1.89E-09	3.91E-08	1.45E-09	0.04	0.40	.00
Language Task	4.66E-08	2.55E-09	3.05E-08	1.19E-09	0.74	19.56***	.17
Relational Task	2.86E-08	1.65E-09	3.14E-08	1.25E-09	0.15	2.47	.03
Social Task	4.07E-08	2.05E-09	3.89E-08	1.37E-09	0.13	1.05	.01
Gambling Task	7.29E-08	4.06E-09	6.23E-08	2.66E-09	0.14	3.94*	.04
Motor Task	7.63E-08	5.21E-09	3.16E-08	1.43E-09	6.49*	81.63***	.46

Note. This table demonstrates means and standard deviations of measures for stability of the stable and instable state, as well as corresponding test statistics of repeated measures ANOVA significance testing. Means and standard deviations are shown with four decimal numbers to increase comparability for the reader.

[a] CV = covariate, here mean difference of activation between the stable and instable state

*** $p < 0.001$, ** $p < 0.01$, * $p < 0.05$

Table S14

Results of repeated measures ANOVA comparing control energy between exclusion of rich club members and a size-matched set of semi-randomly selected regions; analyses based on a binary connectome

Measures	RC excluded		Random excluded		CV	Main effect	
	M	*SD*	*M*	*SD*	*F* (1,96)	*F* (1,96)	η^2
Energy stable -> instable							
Emotion Task	3.20E+07	1.72E+06	4.47E+07	2.30E+06	2.04	15.56***	.14
Working Memory Task	3.95E+07	1.72E+06	3.68E+07	1.39E+06	0.13	2.07	.02
Language Task	3.90E+07	2.12E+06	5.63E+07	1.97E+06	1.34	24.30***	.20
Relational Task	4.96E+07	2.11E+06	4.36E+07	1.98E+06	0.02	5.42*	.05
Social Task	3.97E+07	2.22E+06	4.18E+07	1.81E+06	0.98	0.09	.00
Gambling Task	2.06E+07	1.06E+06	2.29E+07	8.93E+05	0.56	1.69	.02
Motor Task	3.55E+07	2.85E+06	7.65E+07	3.63E+06	2.60	157.89***	.62
Energy instable -> stable							
Emotion Task	2.39E+07	1.49E+06	2.67E+07	1.09E+06	0.18	1.65	.02
Working Memory Task	2.80E+07	1.42E+06	2.62E+07	1.09E+06	1.11	0.21	.00
Language Task	3.78E+07	1.81E+06	5.54E+07	1.79E+06	4.57*	43.26***	.31
Relational Task	3.76E+07	2.43E+06	3.58E+07	2.03E+06	0.25	0.17	.00
Social Task	2.93E+07	1.81E+06	2.76E+07	1.35E+06	11.24**	1.00	.01
Gambling Task	1.90E+07	1.03E+06	2.06E+07	8.48E+05	0.40	0.72	.01
Motor Task	8.49E+06	6.83E+05	1.71E+07	9.18E+05	9.66**	128.64***	.57

Note. This table demonstrates means and standard deviations of measures for control energy for the transition between the stable and instable state, as well as corresponding test statistics of repeated measures ANOVA significance testing. Means and standard deviations are shown with four decimal numbers to increase comparability for the reader.

[a] CV = covariate, here mean difference of activation between the stable and instable state

*** p < 0.001, **p < 0.01, *p < 0.05

Table S15

Results of repeated measures ANOVA comparing state stability between exclusion of individual level rich club members and a size-matched set of semi-randomly selected regions

Measures		RC excluded		Random excluded		CV	Main effect	
		M	*SD*	*M*	*SD*	*F* (1,96)	*F* (1,96)	η^2
Stability stable state								
	Emotion Task	7.81E-08	5.34E-09	4.25E-08	1.53E-09	0.15	20.51***	.18
	Working Memory Task	7.40E-08	6.83E-09	3.75E-08	1.35E-09	0.04	15.21***	.14
	Language Task	5.20E-08	2.57E-09	2.28E-08	8.31E-10	6.19*	112.32***	.54
	Relational Task	4.96E-08	3.60E-09	2.77E-08	9.90E-10	1.62	32.51***	.25
	Social Task	6.71E-08	3.76E-09	4.12E-08	1.71E-09	0.22	44.52***	.32
	Gambling Task	1.01E-07	8.30E-09	5.31E-08	2.38E-09	3.28	32.63***	.25
	Motor Task	1.99E-07	1.18E-08	8.41E-08	3.50E-09	0.08	70.63***	.42
Stability instable state								
	Emotion Task	6.22E-08	3.45E-09	2.98E-08	1.01E-09	0.18	41.81***	.30
	Working Memory Task	5.27E-08	3.65E-09	3.10E-08	1.10E-09	0.39	24.39***	.20
	Language Task	5.40E-08	3.10E-09	2.25E-08	9.15E-10	0.44	66.64***	.41
	Relational Task	4.22E-08	2.64E-09	2.50E-08	9.81E-10	1.75	36.03***	.27
	Social Task	5.65E-08	2.87E-09	3.09E-08	1.07E-09	0.07	67.72***	.41
	Gambling Task	9.08E-08	6.74E-09	5.00E-08	2.19E-09	4.07*	39.25***	.29
	Motor Task	7.63E-08	5.91E-09	2.34E-08	1.16E-09	3.72	70.95***	.43

Note. This table demonstrates means and standard deviations of measures for stability of the stable and instable state, as well as corresponding test statistics of repeated measures ANOVA significance testing. Means and standard deviations are shown with four decimal numbers to increase comparability for the reader.

[a] CV = covariate, here mean difference of activation between the stable and instable state

*** $p < 0.001$, ** $p < 0.01$, * $p < 0.05$

Table S16

Results of repeated measures ANOVA comparing control energy between exclusion of individual level rich club members and a size-matched set of semi-randomly selected regions

Measures	RC excluded		Random excluded		CV	Main effect	
	M	*SD*	*M*	*SD*	*F* (1,96)	*F* (1,96)	η^2
Energy stable -> instable							
Emotion Task	2.79E+07	1.73E+06	5.87E+07	3.30E+06	28.80***	17.49***	.15
Working Memory Task	3.40E+07	2.05E+06	4.63E+07	1.79E+06	2.10	13.26***	.12
Language Task	3.76E+07	1.98E+06	7.58E+07	2.80E+06	4.31*	95.81***	.50
Relational Task	3.93E+07	2.57E+06	5.56E+07	3.06E+06	0.14	16.40***	.15
Social Task	3.23E+07	2.29E+06	5.37E+07	2.53E+06	11.88***	32.71***	.25
Gambling Task	1.99E+07	1.34E+06	2.91E+07	1.18E+06	0.37	28.65***	.23
Motor Task	4.10E+07	3.84E+06	1.08E+08	5.88E+06	1.35	69.23***	.42
Energy instable -> stable							
Emotion Task	2.33E+07	2.03E+06	3.22E+07	1.47E+06	0.19	10.64**	.10
Working Memory Task	2.47E+07	1.87E+06	3.31E+07	1.31E+06	0.59	10.67**	.10
Language Task	3.93E+07	2.20E+06	7.24E+07	2.52E+06	0.12	125.99***	.57
Relational Task	2.92E+07	1.77E+06	4.65E+07	3.14E+06	11.70***	4.43*	.04
Social Task	2.38E+07	1.37E+06	3.41E+07	1.86E+06	10.78**	6.24*	.06
Gambling Task	1.82E+07	1.26E+06	2.64E+07	1.15E+06	1.88	22.10***	.19
Motor Task	9.04E+06	8.12E+05	2.16E+07	1.26E+06	29.50***	69.56***	.42

Note. This table demonstrates means and standard deviations of measures for control energy for the transition between the stable and instable state, as well as corresponding test statistics of repeated measures ANOVA significance testing. Means and standard deviations are shown with four decimal numbers to increase comparability for the reader.

[a] CV = covariate, here mean difference of activation between the stable and instable state

*** p < 0.001, ** p < 0.01, * p < 0.05

Table S17

Results of repeated measures ANOVA comparing state stability between exclusion of individual level rich club members and a size-matched set of semi-randomly selected regions that have been matched regarding similarity of their connectivity profiles

Measures	RC excluded		Random excluded		CV	Main effect	
	M	*SD*	*M*	*SD*	*F* (1,96)	*F* (1,96)	η^2
Stability stable state							
Emotion Task	7.81E-08	5.34E-09	3.76E-08	1.35E-09	0.20	26.79***	.22
Working Memory Task	7.40E-08	6.83E-09	3.49E-08	1.41E-09	0.02	17.89***	.16
Language Task	5.20E-08	2.57E-09	2.37E-08	9.46E-10	6.78*	114.96***	.54
Relational Task	4.96E-08	3.60E-09	2.57E-08	9.54E-10	1.56	38.16***	.28
Social Task	6.71E-08	3.76E-09	3.94E-08	1.62E-09	0.50	49.43***	.34
Gambling Task	1.01E-07	8.30E-09	4.91E-08	2.15E-09	3.57	38.23***	.28
Motor Task	1.99E-07	1.18E-08	8.32E-08	3.58E-09	0.11	76.27***	.44
Stability instable state							
Emotion Task	6.22E-08	3.45E-09	2.79E-08	9.55E-10	0.11	48.59***	.34
Working Memory Task	5.27E-08	3.65E-09	2.96E-08	1.09E-09	0.36	27.85***	.22
Language Task	5.40E-08	3.10E-09	2.37E-08	1.04E-09	0.46	66.92***	.41
Relational Task	4.22E-08	2.64E-09	2.33E-08	8.85E-10	1.52	42.74***	.31
Social Task	5.65E-08	2.87E-09	3.07E-08	1.10E-09	0.17	69.81***	.42
Gambling Task	9.08E-08	6.74E-09	4.61E-08	1.94E-09	3.49	42.91***	.31
Motor Task	7.63E-08	5.91E-09	2.79E-08	1.48E-09	3.47	65.49***	.41

Note. This table demonstrates means and standard deviations of measures for stability of the stable and instable state, as well as corresponding test statistics of repeated measures ANOVA significance testing. Means and standard deviations are shown with four decimal numbers to increase comparability for the reader.

[a] CV = covariate, here mean difference of activation between the stable and instable state

*** $p < 0.001$, ** $p < 0.01$, * $p < 0.05$

Table S18

Results of repeated measures ANOVA comparing control energy between exclusion of individual level rich club members and a size-matched set of semi-randomly selected regions that have been matched regarding similarity of their connectivity profiles

Measures		RC excluded		Random excluded		CV	Main effect	
		M	*SD*	*M*	*SD*	*F* (1,96)	*F* (1,96)	η^2
Energy stable -> instable								
	Emotion Task	2.79E+07	1.73E+06	5.99E+07	3.04E+06	22.44***	35.21***	.27
	Working Memory Task	3.40E+07	2.05E+06	4.76E+07	1.91E+06	1.61	22.96***	.19
	Language Task	3.76E+07	1.98E+06	7.25E+07	2.74E+06	1.98	81.98***	.46
	Relational Task	3.93E+07	2.57E+06	5.80E+07	2.69E+06	0.16	32.53***	.25
	Social Task	3.23E+07	2.29E+06	5.26E+07	2.36E+06	9.30**	56.55***	.37
	Gambling Task	1.99E+07	1.34E+06	3.08E+07	1.27E+06	1.26	40.83***	.30
	Motor Task	4.10E+07	3.84E+06	8.79E+07	5.23E+06	3.86	42.25***	.33
Energy instable -> stable								
	Emotion Task	2.33E+07	2.03E+06	3.63E+07	1.60E+06	0.29	25.33***	.21
	Working Memory Task	2.47E+07	1.87E+06	3.63E+07	1.60E+06	1.42*	24.08***	.20
	Language Task	3.93E+07	2.20E+06	7.01E+07	2.56E+06	0.55	101.86***	.51
	Relational Task	2.92E+07	1.77E+06	4.86E+07	2.78E+06	15.07***	11.83***	.11
	Social Task	2.38E+07	1.37E+06	3.61E+07	2.01E+06	7.78**	11.15**	.10
	Gambling Task	1.82E+07	1.26E+06	2.83E+07	1.31E+06	2.24	34.23***	.26
	Motor Task	9.04E+06	8.12E+05	1.95E+07	1.20E+06	26.78***	63.51***	.40

Note. This table demonstrates means and standard deviations of measures for control energy for the transition between the stable and instable state, as well as corresponding test statistics of repeated measures ANOVA significance testing. Means and standard deviations are shown with four decimal numbers to increase comparability for the reader.

[a] CV = covariate, here mean difference of activation between the stable and instable state

*** p < 0.001, ** p < 0.01, * p < 0.05

Table S19

Results of repeated measures ANOVA comparing state stability between exclusion of individual level rich club members and a size-matched set of semi-randomly selected regions that have been matched regarding total amount of connections

Measures	RC excluded		Random excluded		CV	Main effect	
	M	*SD*	*M*	*SD*	*F* (1,96)	*F* (1,96)	η^2
Stability stable state							
Emotion Task	7.81E-08	5.34E-09	4.83E-08	1.73E-09	0.27	14.71***	.13
Working Memory Task	7.40E-08	6.83E-09	4.29E-08	1.71E-09	0.02	12.13***	.11
Language Task	5.20E-08	2.57E-09	2.94E-08	1.08E-09	6.28*	85.30***	.47
Relational Task	4.96E-08	3.60E-09	3.14E-08	1.19E-09	1.10	23.95***	.20
Social Task	6.71E-08	3.76E-09	4.71E-08	1.98E-09	0.32	29.14***	.23
Gambling Task	1.01E-07	8.30E-09	6.16E-08	2.78E-09	2.87	25.14***	.21
Motor Task	1.99E-07	1.18E-08	1.05E-07	4.25E-09	0.02	53.90***	.36
Stability instable state							
Emotion Task	6.22E-08	3.45E-09	3.51E-08	1.19E-09	0.45	28.44***	.23
Working Memory Task	5.27E-08	3.65E-09	3.59E-08	1.39E-09	0.34	16.82***	.15
Language Task	5.40E-08	3.10E-09	2.85E-08	1.15E-09	0.38	50.49***	.34
Relational Task	4.22E-08	2.64E-09	2.83E-08	1.11E-09	1.15	24.75***	.21
Social Task	5.65E-08	2.87E-09	3.69E-08	1.30E-09	0.23	47.45***	.33
Gambling Task	9.08E-08	6.74E-09	5.74E-08	2.46E-09	2.92	27.46***	.22
Motor Task	7.63E-08	5.91E-09	3.31E-08	1.64E-09	3.06	53.77***	.36

Note. This table demonstrates means and standard deviations of measures for stability of the stable and instable state, as well as corresponding test statistics of repeated measures ANOVA significance testing. Means and standard deviations are shown with four decimal numbers to increase comparability for the reader.

[a] CV = covariate, here mean difference of activation between the stable and instable state

*** $p < 0.001$, ** $p < 0.01$, * $p < 0.05$

Table S20

Results of repeated measures ANOVA comparing control energy between exclusion of individual level rich club members and a size-matched set of semi-randomly selected regions that have been matched regarding total amount of connections

Measures	RC excluded		Random excluded		CV	Main effect	
	M	*SD*	*M*	*SD*	*F* (1,96)	*F* (1,96)	η^2
Energy stable -> instable							
Emotion Task	2.79E+07	1.73E+06	4.77E+07	2.56E+06	36.13***	9.23**	.09
Working Memory Task	3.40E+07	2.05E+06	4.02E+07	1.61E+06	0.97	4.35*	.04
Language Task	3.76E+07	1.98E+06	6.07E+07	2.27E+06	1.57	53.76***	.36
Relational Task	3.93E+07	2.57E+06	4.81E+07	2.27E+06	0.23	9.68**	.09
Social Task	3.23E+07	2.29E+06	4.37E+07	1.95E+06	4.85*	19.50***	.17
Gambling Task	1.99E+07	1.34E+06	2.48E+07	1.03E+06	0.95	7.62**	.07
Motor Task	4.10E+07	3.84E+06	7.39E+07	4.15E+06	0.74	42.11***	.30
Energy instable -> stable							
Emotion Task	2.33E+07	2.03E+06	2.78E+07	1.24E+06	0.19	2.97	.03
Working Memory Task	2.47E+07	1.87E+06	2.95E+07	1.30E+06	0.68	4.09*	.04
Language Task	3.93E+07	2.20E+06	5.72E+07	2.09E+06	0.31	55.56***	.37
Relational Task	2.92E+07	1.77E+06	3.98E+07	2.34E+06	11.91***	1.91	.02
Social Task	2.38E+07	1.37E+06	2.99E+07	1.59E+06	5.66*	2.47	.03
Gambling Task	1.82E+07	1.26E+06	2.24E+07	9.59E+05	0.29	7.56**	.07
Motor Task	9.04E+06	8.12E+05	1.57E+07	9.51E+05	11.40**	50.52***	.34

Note. This table demonstrates means and standard deviations of measures for control energy for the transition between the stable and instable state, as well as corresponding test statistics of repeated measures ANOVA significance testing. Means and standard deviations are shown with four decimal numbers to increase comparability for the reader.

[a] CV = covariate, here mean difference of activation between the stable and instable state

*** $p < 0.001$, ** $p < 0.01$, * $p < 0.05$

Table S21

Results of repeated measures ANOVA comparing control energy between exclusion of rich club members and a size-matched set of semi-randomly selected regions when transitioning between states belonging to different tasks

From:		RC excluded		Random excluded		CV	Main effect	
		M	*SD*	*M*	*SD*	*F* (1,96)	*F* (1,96)	η^2
Emotion Task, to:								
	Working Memory Task	5.71E+07	2.82E+06	6.62E+07	2.64E+06	0.16	6.84*	.07
	Language Task	8.03E+07	5.11E+06	1.52E+08	5.91E+06	0.15	82.38***	.46
	Relational Task	7.54E+07	3.30E+06	8.16E+07	3.81E+06	0.12	0.56	.01
	Social Task	5.71E+07	2.83E+06	7.14E+07	3.23E+06	13.80***	0.65	.01
	Gambling Task	3.02E+07	1.65E+06	4.10E+07	1.63E+06	0.62	28.38***	.23
	Motor Task	2.70E+07	1.71E+06	5.26E+07	2.28E+06	7.13**	76.00***	.44
Working Memory Task, to:								
	Emotion Task	4.21E+07	2.89E+06	7.21E+07	4.80E+06	25.73***	14.47***	.13
	Language Task	8.75E+07	5.19E+06	1.59E+08	5.66E+06	3.60	26.74***	.33
	Relational Task	6.53E+07	3.27E+06	7.31E+07	5.13E+06	16.25***	3.16	.03
	Social Task	5.25E+07	2.94E+06	7.10E+07	3.32E+06	12.07***	3.69	.04
	Gambling Task	2.10E+07	1.54E+06	3.14E+07	1.34E+06	0.66	67.06***	.41
	Motor Task	2.80E+07	1.45E+06	5.13E+07	1.99E+06	0.91	48.64***	.34
Language Task, to:								
	Emotion Task	6.57E+07	4.14E+06	1.25E+08	6.58E+06	26.81***	17.29***	.15
	Working Memory Task	8.85E+07	4.09E+06	1.26E+08	4.20E+06	1.46	14.40***	.13
	Relational Task	1.16E+08	4.87E+06	1.52E+08	6.04E+06	1.10	5.31*	.05
	Social Task	7.86E+07	3.88E+06	1.22E+08	4.72E+06	10.24***	3.34	.03
	Gambling Task	5.65E+07	2.61E+06	8.97E+07	3.03E+06	1.46	28.29***	.23
	Motor Task	2.84E+07	1.68E+06	5.76E+07	2.24E+06	0.17	68.29***	.42
Relational Task, to:								
	Emotion Task	3.81E+07	1.98E+06	6.56E+07	3.52E+06	0.49	28.33***	.23
	Working Memory Task	4.28E+07	2.18E+06	5.12E+07	2.36E+06	0.13	7.70**	.07
	Language Task	9.24E+07	5.28E+06	1.63E+08	5.44E+06	3.01	76.46***	.44
	Social Task	4.76E+07	2.48E+06	7.06E+07	2.89E+06	19.70***	10.26**	.01
	Gambling Task	2.07E+07	1.25E+06	3.15E+07	1.40E+06	0.03	47.05***	.33
	Motor Task	3.44E+07	1.74E+06	5.56E+07	2.09E+06	0.03	51.75***	.35
Social Task, to:								
	Emotion Task	4.57E+07	2.81E+06	7.51E+07	4.46E+06	3.35	24.22***	.20
	Working Memory Task	5.55E+07	2.64E+06	6.88E+07	2.55E+06	0.76	6.98**	.07
	Language Task	8.18E+07	4.87E+06	1.51E+08	5.24E+06	0.44	55.47***	.37
	Relational Task	7.32E+07	3.22E+06	9.04E+07	4.21E+06	1.61	4.21*	.04
	Gambling Task	3.31E+07	1.81E+06	4.58E+07	1.64E+06	0.13	26.46***	.22
	Motor Task	2.99E+07	1.66E+06	5.20E+07	2.09E+06	2.04	42.63***	.31
Gambling Task, to:								
	Emotion Task	4.67E+07	2.84E+06	7.69E+07	4.86E+06	33.56***	10.70**	.10
	Working Memory Task	5.22E+07	2.41E+06	6.13E+07	2.37E+06	1.13	3.67	.04
	Language Task	8.60E+07	5.00E+06	1.52E+08	5.36E+06	1.91	69.69***	.42
	Relational Task	7.47E+07	3.52E+06	8.32E+07	5.06E+06	8.32**	0.80	.01
	Social Task	6.06E+07	3.23E+06	7.79E+07	3.34E+06	13.16***	0.62	.01
	Motor Task	2.34E+07	1.24E+06	4.06E+07	1.71E+06	0.25	57.26***	.37
Motor Task, to:								
	Emotion Task	6.61E+07	3.77E+06	1.09E+08	5.88E+06	24.16***	11.89***	.11
	Working Memory Task	8.12E+07	3.50E+06	1.01E+08	3.60E+06	1.81	5.04*	.05
	Language Task	8.02E+07	5.06E+06	1.40E+08	5.06E+06	1.01	73.54***	.43
	Relational Task	1.10E+08	4.74E+06	1.27E+08	5.91E+06	2.98	0.43	.00
	Social Task	8.02E+07	4.05E+06	1.04E+08	4.37E+06	13.12***	0.46	.00
	Gambling Task	4.59E+07	2.23E+06	6.07E+07	2.24E+06	0.27	22.11***	.19

Note. This table demonstrates means and standard deviations of measures for control energy for the transition between two states belonging to different tasks, as well as corresponding test statistics of repeated measures ANOVA significance testing. Means and standard deviations are rounded to increase comparability for the reader.

[a] CV = covariate, here mean difference of activation between state A and state B

*** $p < 0.001$, ** $p < 0.01$, * $p < 0.05$

Table S22

Mean rank of regional contribution per resting state network and over all stability and energy measures, respectively

	stability		energy	
	$M_{rank(emp)}$	$M_{rank(rand)}$	$M_{rank(emp)}$	$M_{rank(rand)}$
Yeo 1 – central visual	60.10***	109.67	67.65***	109.82
Yeo 2 – peripheral visual	103.47	110.23	106.34	110.22
Yeo 3 – dorsal somatomotor	132.43*	109.89	131.77**	109.85
Yeo 4 – ventral somatomotor	105.03	110.07	105.83	110.05
Yeo 5 – posterior dorsal attention	103.99	109.88	108.05	109.88
Yeo 6 – somatomotor association	118.44	110.06	119.02	110.02
Yeo 7 – posterior ventral attention	117.77	109.88	117.97	109.89
Yeo 8 – anterior ventral attention	114.62	109.76	111.56	109.86
Yeo 9 – medial temporal-limbic	119.81	110.15	116.96	110.15
Yeo 10 – orbitofrontal-limbic	98.18	110.08	93.58	110.05
Yeo 11 – posterior frontoparietal	137.47*	109.88	135.76*	109.82
Yeo 12 – ventro-lateral frontoparietal	91.73*	110.24	93.67*	110.17
Yeo 13 – dorso-lateral frontoparietal	124.21*	109.69	119.73	109.82
Yeo 14 – lateral temporal DMN	113.65	109.80	114.21	109.91
Yeo 15 – ventral DMN	117.22	110.62	116.09	110.40
Yeo 16 – dorsal DMN	113.08	110.20	112.80	110.14
Yeo 17 – lateral DMN	117.55	110.27	114.37	110.21

Note. This table demonstrates the mean empirical rank per resting state network regarding regional contribution to stability and energy measures; empirical values were compared to a null distribution in which regional rank was permutated 10000-fold before calculating the mean per network. Significance values are based on this comparison, and the mean randomized rank per network is included here to indicate direction of effect. Means and standard deviations are rounded to increase comparability for the reader.

DMN = default mode network

*** $p < 0.001$, ** $p < 0.01$, * $p < 0.05$

Table S23

Regions in the Lausanne parcellation ordered as to reflect their mean rank in regional stability & control energy analyses; most highly ranked region (i.e. region with the biggest control contribution) first

Order	according to mean rank in stability measures	according to mean rank in energy measures
1	lh-lateraloccipital_3	rh-medialorbitofrontal_3
2	lh-fusiform_1	lh-lateraloccipital_3
3	lh-superiorparietal_3	lh-lateralorbitofrontal_1
4	rh-lingual_1	lh-medialorbitofrontal_1
5	lh-medialorbitofrontal_1	lh-fusiform_1
6	lh-fusiform_2	lh-superiorparietal_3
7	rh-fusiform_2	lh-insula_4
8	rh-fusiform_1	lh-lingual_3
9	rh-lateraloccipital_3	lh-frontalpole_1
10	lh-lateraloccipital_5	lh-fusiform_2
11	rh-medialorbitofrontal_3	rh-fusiform_1
12	lh-insula_4	rh-caudalmiddlefrontal_2
13	lh-lingual_1	rh-lateraloccipital_3
14	rh-lateraloccipital_4	lh-lateralorbitofrontal_3
15	lh-lateraloccipital_2	lh-postcentral_6
16	lh-lingual_3	rh-fusiform_2
17	rh-lingual_2	rh-lateralorbitofrontal_1
18	rh-postcentral_1	lh-caudalmiddlefrontal_2
19	rh-pericalcarine_2	rh-rostralmiddlefrontal_3
20	rh-caudalmiddlefrontal_1	lh-rostralmiddlefrontal_1
21	rh-lateraloccipital_2	rh-parstriangularis_1
22	rh-precuneus_2	rh-lateralorbitofrontal_3
23	rh-medialorbitofrontal_1	lh-lateraloccipital_2
24	rh-insula_1	rh-lingual_2
25	rh-superiorparietal_6	rh-lingual_1
26	rh-supramarginal_1	rh-postcentral_1
27	lh-lateralorbitofrontal_3	rh-rostralmiddlefrontal_4
28	rh-parstriangularis_1	lh-lateraloccipital_5
29	lh-caudalmiddlefrontal_2	lh-lingual_4
30	lh-postcentral_6	rh-frontalpole_1
31	lh-fusiform_3	rh-superiorparietal_6
32	rh-rostralmiddlefrontal_3	lh-lingual_2
33	rh-caudalmiddlefrontal_2	lh-rostralmiddlefrontal_3
34	lh-frontalpole_1	rh-lateraloccipital_2
35	lh-rostralanteriorcingulate_1	rh-medialorbitofrontal_2
36	lh-precentral_7	rh-caudalmiddlefrontal_1
37	lh-lingual_2	lh-rostralanteriorcingulate_1
38	lh-lateralorbitofrontal_1	lh-superiorfrontal_4
39	lh-superiorfrontal_4	rh-lateraloccipital_4
40	lh-precentral_5	rh-rostralmiddlefrontal_2
41	rh-rostralmiddlefrontal_2	lh-fusiform_3
42	rh-lateralorbitofrontal_3	lh-lingual_1
43	rh-middletemporal_4	rh-medialorbitofrontal_1
44	rh-superiorparietal_4	rh-superiorparietal_4
45	lh-rostralmiddlefrontal_3	lh-medialorbitofrontal_2
46	lh-lingual_4	rh-supramarginal_1
47	lh-supramarginal_5	rh-cuneus_1
48	lh-middletemporal_4	rh-lateralorbitofrontal_4
49	lh-rostralmiddlefrontal_1	rh-insula_1
50	lh-precuneus_5	rh-precuneus_2
51	rh-lateralorbitofrontal_1	rh-pericalcarine_2
52	rh-pericalcarine_1	lh-inferiorparietal_3
53	lh-lateraloccipital_4	rh-superiorfrontal_8
54	rh-parsopercularis_2	rh-pericalcarine_1
55	rh-precentral_2	lh-rostralmiddlefrontal_2
56	rh-rostralmiddlefrontal_4	lh-superiorfrontal_2

57	rh-cuneus_1	lh-lateralorbitofrontal_2
58	rh-lateraloccipital_5	lh-posteriorcingulate_2
59	lh-superiorfrontal_1	lh-transversetemporal_1
60	rh-supramarginal_4	lh-superiorfrontal_1
61	rh-transversetemporal_1	rh-transversetemporal_1
62	lh-rostralmiddlefrontal_2	rh-lateraloccipital_5
63	rh-rostralanteriorcingulate_1	rh-rostralanteriorcingulate_1
64	rh-inferiortemporal_4	rh-middletemporal_4
65	lh-inferiorparietal_3	lh-precentral_7
66	lh-superiorparietal_6	rh-postcentral_2
67	rh-lateraloccipital_1	rh-rostralmiddlefrontal_5
68	rh-frontalpole_1	lh-bankssts_2
69	rh-superiortemporal_4	lh-supramarginal_5
70	rh-precentral_1	lh-middletemporal_4
71	lh-lateraloccipital_1	rh-inferiortemporal_4
72	lh-precuneus_4	lh-supramarginal_1
73	lh-isthmuscingulate_1	lh-lateralorbitofrontal_4
74	rh-rostralmiddlefrontal_5	rh-precentral_2
75	lh-transversetemporal_1	lh-superiorparietal_6
76	rh-lateralorbitofrontal_4	rh-parsopercularis_2
77	rh-temporalpole_1	lh-postcentral_4
78	rh-medialorbitofrontal_2	rh-inferiorparietal_5
79	rh-superiortemporal_5	lh-lateraloccipital_4
80	rh-insula_2	rh-entorhinal_1
81	lh-posteriorcingulate_2	rh-supramarginal_4
82	lh-postcentral_4	lh-precentral_5
83	rh-inferiorparietal_5	lh-rostralmiddlefrontal_6
84	rh-fusiform_3	lh-rostralmiddlefrontal_4
85	lh-superiorfrontal_2	rh-inferiortemporal_3
86	lh-superiortemporal_4	rh-postcentral_4
87	lh-pericalcarine_1	lh-precuneus_5
88	lh-inferiortemporal_1	rh-precentral_1
89	lh-superiortemporal_5	rh-fusiform_3
90	lh-parsorbitalis_1	lh-precentral_6
91	lh-precentral_6	rh-temporalpole_1
92	lh-rostralmiddlefrontal_6	lh-superiorfrontal_3
93	rh-inferiortemporal_1	lh-isthmuscingulate_1
94	lh-medialorbitofrontal_2	rh-middletemporal_3
95	lh-insula_1	rh-lateralorbitofrontal_2
96	lh-supramarginal_1	lh-parsorbitalis_1
97	rh-postcentral_2	rh-rostralmiddlefrontal_1
98	lh-lateralorbitofrontal_2	rh-bankssts_1
99	rh-superiorfrontal_8	rh-inferiortemporal_1
100	lh-caudalmiddlefrontal_1	lh-temporalpole_1
101	rh-postcentral_3	lh-rostralmiddlefrontal_5
102	rh-precentral_5	lh-inferiortemporal_1
103	lh-bankssts_2	lh-caudalanteriorcingulate_1
104	rh-bankssts_1	lh-lateraloccipital_1
105	rh-precuneus_3	lh-postcentral_7
106	lh-temporalpole_1	lh-caudalmiddlefrontal_1
107	lh-supramarginal_2	rh-lateraloccipital_1
108	rh-middletemporal_3	rh-rostralmiddlefrontal_6
109	rh-postcentral_4	rh-superiortemporal_4
110	rh-superiorparietal_7	rh-superiortemporal_2
111	lh-postcentral_5	rh-inferiortemporal_2
112	lh-superiorfrontal_3	lh-entorhinal_1
113	rh-superiortemporal_3	lh-supramarginal_2
114	rh-superiortemporal_1	lh-insula_1
115	lh-caudalanteriorcingulate_1	lh-pericalcarine_1
116	lh-superiortemporal_2	rh-superiortemporal_5

117	lh-bankssts_1	lh-parsopercularis_1
118	rh-entorhinal_1	lh-bankssts_1
119	lh-superiorparietal_2	rh-parsopercularis_1
120	rh-rostralmiddlefrontal_6	lh-parahippocampal_1
121	lh-middletemporal_3	lh-superiorparietal_2
122	lh-rostralmiddlefrontal_4	lh-postcentral_5
123	lh-lateralorbitofrontal_4	rh-precentral_5
124	rh-cuneus_2	rh-superiorfrontal_1
125	lh-inferiorparietal_1	lh-superiortemporal_5
126	rh-parstriangularis_2	rh-lingual_3
127	rh-superiortemporal_2	lh-superiortemporal_2
128	rh-inferiortemporal_3	lh-precuneus_4
129	rh-inferiorparietal_3	rh-precuneus_3
130	rh-inferiorparietal_6	lh-inferiortemporal_3
131	lh-postcentral_7	rh-parstriangularis_2
132	rh-lateralorbitofrontal_2	rh-middletemporal_2
133	rh-isthmuscingulate_1	lh-supramarginal_3
134	rh-superiorfrontal_4	rh-cuneus_2
135	lh-inferiortemporal_4	rh-superiortemporal_1
136	lh-supramarginal_3	rh-postcentral_3
137	rh-rostralmiddlefrontal_1	lh-superiorfrontal_6
138	rh-parsorbitalis_1	rh-paracentral_3
139	rh-parsopercularis_1	rh-superiortemporal_3
140	lh-insula_2	rh-parsorbitalis_1
141	lh-superiorparietal_7	rh-superiorparietal_1
142	lh-caudalmiddlefrontal_3	rh-insula_2
143	rh-inferiortemporal_2	rh-paracentral_2
144	rh-superiorfrontal_5	rh-superiorfrontal_4
145	lh-parahippocampal_1	lh-inferiortemporal_4
146	rh-superiorfrontal_1	rh-isthmuscingulate_1
147	rh-inferiorparietal_1	rh-inferiorparietal_3
148	lh-entorhinal_1	lh-superiortemporal_4
149	lh-postcentral_2	lh-middletemporal_3
150	lh-parstriangularis_1	lh-caudalmiddlefrontal_3
151	rh-paracentral_3	rh-caudalanteriorcingulate_1
152	lh-rostralmiddlefrontal_5	rh-paracentral_1
153	rh-lingual_3	lh-posteriorcingulate_1
154	lh-parsopercularis_1	rh-parahippocampal_1
155	rh-posteriorcingulate_2	rh-inferiorparietal_1
156	rh-supramarginal_3	rh-superiorfrontal_7
157	rh-middletemporal_2	rh-superiorparietal_7
158	lh-superiorfrontal_6	rh-inferiorparietal_6
159	rh-precentral_3	rh-precentral_3
160	rh-caudalanteriorcingulate_1	lh-inferiorparietal_1
161	lh-inferiorparietal_5	lh-fusiform_4
162	rh-postcentral_5	lh-paracentral_1
163	lh-posteriorcingulate_1	lh-parstriangularis_1
164	rh-paracentral_2	rh-insula_3
165	lh-superiorparietal_4	lh-inferiortemporal_2
166	lh-precentral_4	lh-inferiorparietal_5
167	lh-inferiorparietal_4	lh-precentral_4
168	rh-superiorparietal_5	lh-precentral_1
169	lh-insula_3	lh-precuneus_2
170	lh-superiorfrontal_8	rh-posteriorcingulate_1
171	rh-parahippocampal_1	rh-superiorfrontal_5
172	rh-insula_3	lh-postcentral_2
173	rh-superiorfrontal_7	rh-posteriorcingulate_2
174	rh-precuneus_1	lh-insula_2
175	lh-cuneus_1	rh-superiorfrontal_2
176	lh-precentral_8	lh-precentral_8

177	lh-middletemporal_1	rh-supramarginal_3
178	lh-inferiortemporal_2	rh-caudalmiddlefrontal_3
179	rh-paracentral_1	lh-middletemporal_2
180	lh-middletemporal_2	lh-inferiorparietal_2
181	lh-precuneus_2	rh-superiorparietal_5
182	lh-inferiorparietal_2	rh-fusiform_4
183	lh-precentral_2	lh-precentral_2
184	lh-superiorparietal_5	lh-superiorparietal_5
185	rh-superiorparietal_1	lh-superiorparietal_7
186	lh-postcentral_3	lh-insula_3
187	rh-posteriorcingulate_1	rh-superiorfrontal_3
188	lh-superiortemporal_1	lh-superiorfrontal_9
189	lh-superiorfrontal_7	rh-precuneus_1
190	lh-precentral_1	lh-inferiorparietal_4
191	rh-superiorparietal_2	lh-superiorparietal_4
192	lh-superiortemporal_3	lh-superiortemporal_3
193	lh-paracentral_1	lh-cuneus_1
194	rh-inferiorparietal_4	lh-paracentral_2
195	rh-inferiorparietal_2	lh-postcentral_3
196	lh-inferiortemporal_3	lh-superiortemporal_1
197	lh-superiorparietal_1	lh-middletemporal_1
198	lh-fusiform_4	rh-inferiorparietal_4
199	rh-caudalmiddlefrontal_3	lh-parsopercularis_2
200	rh-superiorfrontal_3	lh-superiorfrontal_8
201	lh-superiorfrontal_9	rh-inferiorparietal_2
202	rh-fusiform_4	lh-superiorfrontal_7
203	lh-parsopercularis_2	rh-superiorfrontal_6
204	rh-precentral_4	rh-precentral_4
205	rh-superiorfrontal_6	lh-superiorfrontal_5
206	lh-postcentral_1	lh-postcentral_1
207	rh-superiorparietal_3	rh-superiorparietal_2
208	rh-superiorfrontal_2	lh-precentral_3
209	rh-middletemporal_1	rh-middletemporal_1
210	lh-precuneus_1	lh-superiorparietal_1
211	lh-superiorfrontal_5	rh-superiorparietal_3
212	lh-precentral_3	rh-supramarginal_2
213	rh-supramarginal_2	lh-precuneus_1
214	lh-paracentral_2	rh-postcentral_5
215	lh-precuneus_3	lh-precuneus_3
216	rh-precuneus_4	rh-precentral_6
217	lh-supramarginal_4	rh-precuneus_4
218	rh-precuneus_5	lh-supramarginal_4
219	rh-precentral_6	rh-precuneus_5

Table S24

Results of repeated measures ANOVA comparing state stability between exclusion of rich club members and a size-matched set of semi-randomly selected regions; HCP-MMP parcellation

Measures		RC excluded		Random excluded		CV	Main effect	
		M	*SD*	*M*	*SD*	*F* (1,96)	*F* (1,96)	η^2
Stability stable state								
	Emotion Task	4.45E-08	5.61E-09	2.90E-09	2.38E-10	2.92	42.06***	0.31
	Working Memory Task	4.80E-08	3.72E-09	2.99E-09	3.03E-10	0.51	82.82***	0.47
	Language Task	1.38E-08	1.15E-09	1.20E-09	1.14E-10	2.70	36.99***	0.29
	Relational Task	4.49E-08	5.55E-09	1.92E-09	1.83E-10	0.50	36.54***	0.28
	Social Task	5.86E-08	5.36E-09	1.91E-09	1.74E-10	2.68	80.83***	0.47
	Gambling Task	5.43E-08	4.30E-09	3.89E-09	4.28E-10	8.30**	111.76***	0.55
	Motor Task	7.43E-08	6.37E-09	5.61E-09	4.00E-10	0.01	56.34***	0.38
Stability instable state								
	Emotion Task	2.98E-08	2.75E-09	2.01E-09	1.79E-10	3.38	73.73***	0.44
	Working Memory Task	4.05E-08	3.07E-09	2.32E-09	1.97E-10	6.33*	116.34***	0.56
	Language Task	1.07E-08	1.11E-09	1.28E-09	1.10E-10	0.31	35.58***	0.28
	Relational Task	3.30E-08	2.72E-09	1.75E-09	1.65E-10	1.91	88.14***	0.49
	Social Task	3.53E-08	2.90E-09	1.51E-09	1.38E-10	5.21*	107.38***	0.54
	Gambling Task	5.60E-08	4.01E-09	3.66E-09	4.25E-10	4.87*	116.49***	0.56
	Motor Task	2.31E-08	1.89E-09	2.01E-09	1.48E-10	2.12	81.74***	0.47

Note. This table demonstrates means and standard deviations of measures for stability of the instable and stable state, as well as corresponding test statistics of repeated measures ANOVA significance testing. Means and standard deviations are rounded to increase comparability for the reader.

[a] CV = covariate, here mean difference of activation between the instable and stable state

*** $p < 0.001$, ** $p < 0.01$, * $p < 0.05$

Table S25

Results of repeated measures ANOVA comparing state stability between exclusion of rich club members and a size-matched set of semi-randomly selected regions; HCP-MMP parcellation

Measures		RC excluded		Random excluded		CV	Main effect	
		M	*SD*	*M*	*SD*	*F* (1,96)	*F* (1,96)	η^2
Energy A -> B								
	Emotion Task	1.37E+08	2.26E+07	1.51E+09	1.85E+08	4.42*	16.59***	0.15
	Working Memory Task	6.84E+07	8.54E+06	1.28E+09	1.71E+08	0.19	20.65***	0.18
	Language Task	4.16E+08	6.88E+07	1.93E+09	2.00E+08	0.34	32.24***	0.26
	Relational Task	8.29E+07	1.28E+07	1.42E+09	1.51E+08	0.07	37.94***	0.29
	Social Task	8.95E+07	1.02E+07	2.21E+09	4.01E+08	3.15	7.23**	0.07
	Gambling Task	4.45E+07	4.90E+06	7.73E+08	9.84E+07	0.15	27.79***	0.23
	Motor Task	1.66E+08	2.00E+07	1.77E+09	1.88E+08	23.78***	10.38**	0.10
Energy B -> A								
	Emotion Task	5.52E+07	7.67E+06	7.92E+08	9.09E+07	0.72	25.54***	0.22
	Working Memory Task	5.34E+07	6.54E+06	9.90E+08	1.75E+08	0.02	14.31***	0.13
	Language Task	1.89E+08	3.53E+07	2.25E+09	2.75E+08	1.19	35.72***	0.28
	Relational Task	8.74E+07	2.26E+07	1.29E+09	1.82E+08	1.48	17.47***	0.16
	Social Task	5.95E+07	1.26E+07	1.42E+09	2.78E+08	5.78*	4.21*	0.04
	Gambling Task	5.59E+07	1.18E+07	6.92E+08	7.91E+07	0.22	32.18***	0.26
	Motor Task	3.79E+07	4.23E+06	4.51E+08	7.65E+07	9.79**	2.57	0.03

Note. This table demonstrates means and standard deviations of measures for stability of the instable and stable state, as well as corresponding test statistics of repeated measures ANOVA significance testing. Means and standard deviations are rounded to increase comparability for the reader.

[a] CV = covariate, here mean difference of activation between the instable and stable state

*** $p < 0.001$, ** $p < 0.01$, * $p < 0.05$
