# Supplementary material for: Exploring the Role of the Rich Club in Network Control of Neurocognitive States
Source: Hum Brain Mapp. 2026 Feb 26;47(4):e70485. doi: 10.1002/hbm.70485 (PMC12945927; doi:10.1002/hbm.70485)
Supplement: Supplementary file 2 — Data S2: hbm70485‐sup‐0002‐Supinfo2.pdf. [file HBM-47-e70485-s001.pdf]

Supplement: Exploring the role of the rich club in network control of neurocognitive states

Alina N. Podschun *[1,2], Richard F. Betzel[3], Urs Braun[4], and Sebastian Markett[1]

[1]Humboldt-Universität zu Berlin, Berlin, Germany
[2]International Psychoanalytic University Berlin, Berlin, Germany
[3]University of Minnesota, Twin Cities, Minneapolis, MN, USA
[4]Zentralinstitut für Seelische Gesundheit in Mannheim, Mannheim, Germany

November 25, 2025

1 Supplemental methods & results

1.1 HCP acquisition pipeline and processing

Details regarding the acquisition pipeline can be found in Van Essen et al. (2013) and Glasser et al. (2013). All MRI data was obtained on a Siemens 3T scanner with a 32 channel head coil.

Regarding diffusion imaging, high-resolution diffusion weighted images (1.25mm isotropic) were obtained using a Stejskal-Tanner (monopolar) diffusion-encoding scheme with a 100 mT/m gradient set and multiband factor of 3, achieving sufficient SNR at 1.25mm resolution with diffusion-weighting up to b=3000 s/mm^2. Distortions from eddy currents were addressed by acquiring diffusion data in six series with reversed phase-encoding directions (RL and LR) within each pair. T1- as well as T2-weighted anatomical scans were acquired at resolution of 0.7mm isotropic. The preprocessing pipeline began with intensity normalization of the mean b0 image across the six series. EPI distortions were estimated using FSL's "topup" tool, followed by eddy-current and motion correction with FSL's "eddy" tool. Gradient nonlinearity corrections were applied after running eddy. The corrected diffusion data were then resampled into 1.25mm native structural space and masked. Finally, the corrected b0 image was registered to the T1-weighted structural image using boundary-based registration, transforming the diffusion data to structural space for accurate fiber orientation estimation. Preprocessing analyses relied on FreeSurfer 5.1. (Glasser et al., 2013).

Regarding functional MRI data, grayordinate-based, MSM-All registered results of within-participant taskfMRI analyses were used as provided by the HCP. Data was acquired with a multiband factor of 8, TR = 720ms and a voxel size of 2mm isotropic (Van Essen et al., 2013), preprocessed by the consortium itself using a minimal preprocessing pipeline, including gradient unwarping, smoothing to a level of 2mm, motion and distortion correction, registration to MNI152 space and grand-mean

*Corresponding author email: podschal@hu-berlin.de

intensity normalization (Barch et al., 2013; Glasser et al., 2013). Subsequent within-participant analysis by the HCP included high-pass filtering at 200s and a fixed-effect analysis conducted with the FMRIB Software Library (FSL; Jenkinson et al., 2012) the output is in standard HCP grayordinate space in CIFTI format (Glasser et al., 2013; Van Essen et al., 2013, also for further details).

1.2 Closer description of task paradigms used by the HCP

We used data of all 7 behaviorally constricted tasks acquired by the HCP, namely an emotion matching task, a version of an n-back working memory paradigm, a gambling task for investigation of incentive processing, a language task comparing language to mathematical processing, a motor task eliciting movement, a relational task asking participants to match visual properties of objects, and a social task assessing whether participants thought of objects having a random or social interaction. The 7 tasks provided in the HCP have been chosen with a focus on application to connectivity research and on grounds of their established adequate psychometric criteria, as well as because of their combined ability to investigate a wide array of cognitive functions and activate widespread regions of the brain (Barch et al., 2013). Short descriptions as follows are adapted from information in Barch et al. (2013).

In the emotion task, participants viewed faces displaying different emotions (fearful or neutral) and shapes. Two faces – or objects – were presented at the bottom of the screen, and participants asked to indicate which of the two matches a face or object presented at the top of the screen. Participants were informed through a cue word about which of the two conditions would be following. The task is known to be moderately reliable across time.

The working memory task involved a 2-back and 0-back working memory challenge. Participants were shown blocks of pictures (faces, places, tools, and body parts) and had to indicate if the current image matched one shown two images earlier (2-back) or the currently shown target image (0-back). This task assesses both working memory and category-specific representations. For our investigation, we focused on the working memory aspect of the task, basing brain states category-agnostic on the 0-back and 2-back conditions.

In the gambling task, participants must guess whether a number on a visually presented card is greater or smaller than five to win or lose money. Trials could be rewards, losses, or neutral (with the target number equaling five) and were followed by immediate feedback. The task includes reward blocks (with frequent wins) and loss blocks (with frequent losses). This paradigm is reliably associated with activity in the striatum and other reward-processing brain regions.

The language task contrasts responses when actively listening to a story versus listening to and solving arithmetic problems. The math condition was included as a control since it is unlikely to activate regions involved in semantic processing, and thus constitutes a suitable baseline. In the story condition, participants listened to brief stories and answered forced-choice comprehension questions, with auditory input lasting for 5-9 sentences; in the math condition, participants listened to and solved short arithmetic problems.

To study motion processing, participants performed movements in response to visual cues, including moving their left or right foot, left or right hand, or tongue. Task activity maps on primary motor areas. The relational processing task required participants to compare pairs of variably shaped and textured objects, judging whether the top and bottom pair match in certain relationships. The

task alternated between relational and control conditions, with the relational condition requiring participants to first indicate the dimension of difference between two objects presented at the top, and then judge whether a bottom object pair matches this difference. In the control condition, only one object was presented at the bottom, and participants asked to judge whether this object matched either of the top objects on a previously indicated dimension.

The social cognition paradigm involved watching 20-second videos of objects interacting in ways that imply social interaction (mental condition) versus random movement (random condition). Participants then judged whether the interactions were intentional, not intentional, or whether this was unclear. This task is designed to map onto brain areas involved in mentalization.

1.3 Replication of results a different parcellation scheme

We replicated group-level analyses of the rich club's role in control processes on an alternative parcellation scheme, namely the 358-region multimodal parcellation provided with the Human Connectome Project (excluding both hippocampi from the originally distributed 360-region version). This parcellation schmeme was chosen based on it being widely used (Yan et al., 2023), its biological plausibility (Van Essen et al., 2013) and its relative closeness in resolution to the Lausanne parcellation we used in our main analyses. As this parcellation sheme is not inherent within CATO, we extended our replications to a different toolbox, reconstructing connectomes in MRtrix3. Preprocessed diffusion weighted images were bias corrected, and constrained spherical deconvolution (CSD) applied to estimate fiber orientation distributions. Anatomically constrained deterministic tractography was then performed using the fiber assignment by continuous tracting (FACT) algorithm, generating streamlines seeded throughout the white matter. 10 Million streamlines were selected. Fibers were disregarded if they were less than 5mm or more than 300mm long, and streamlines terminated when reaching boundaries of different tissue types and when the FOD amplitude became smaller than a cutoff value of 0.06. The parcellation as provided by the Human Connectome Project was mapped onto participant-specific surfaces using freesurfer functions and then converted to volume space and registered to diffusion space to be compatible with MRtrix3 processing. These participant-specific parcellations were then used to define brain regions and derive connectomes weighted by the number of streamlines (NOS) connecting two regions.

Findings remain equivalent to those reported in the main text: The rich club was never significantly more involved in control of dynamics than a size-matched reference set. In the overwhelming amount of cases (namely all stability measures and all but one state transition), the rich club indeed was significantly less involved in control processes than the reference set. Detailed results can be seen in Supplemental Figure 5 and Supplemental Tables S24-S25.

1.4 Replication of results on resting-state derived brain states

To increase ecological validity of our findings, we replicated our main investigations regarding a possible control role of the rich club as a network on resting-state derived functional brain states. We based this analysis on 15 group-level intrinsic connectivity networks (ICNs) as derived via independent component analysis (ICA) and provided by the Human Connectome Project. We then employed a dual-regression approach via workbench command to map group-level ICNs back onto the participant level, firstly regressing the group-level ICNs against individuals resting state data (smoothed to a level of 4mm) and thus retrieving individualized ICN-specific time series. In a second step, we conducted multivariate temporal regression on the participant-specific and ICN-specific timecourses

to estimate participant-specific and ICN-specific spatial maps. We then used these spatial maps as our participant-specific resting-state derived brain states, and investigated the rich club's role in controlling dynamics between all pairs of these brain states, as described in the main text.

We found that for 209 out of 210 possible pairs of resting-state derived brain states, the rich club significantly did not contribute more to control of state traversals than a size-matched set of semi-randomly selected regions. In detail, the rich club was significantly more involved in controlling transitions between 1 pair of resting-state derived brain states; not significantly differently involved from a reference set in 71 pairs of brain states, and significantly less involved in control than the reference set for the remaining 138 pairs of brain states. Effect sizes for these analyses are visualized in Supplemental Figure 6. This result overwhelmingly supports the findings detailed in the main text.

2 Supplemental figures

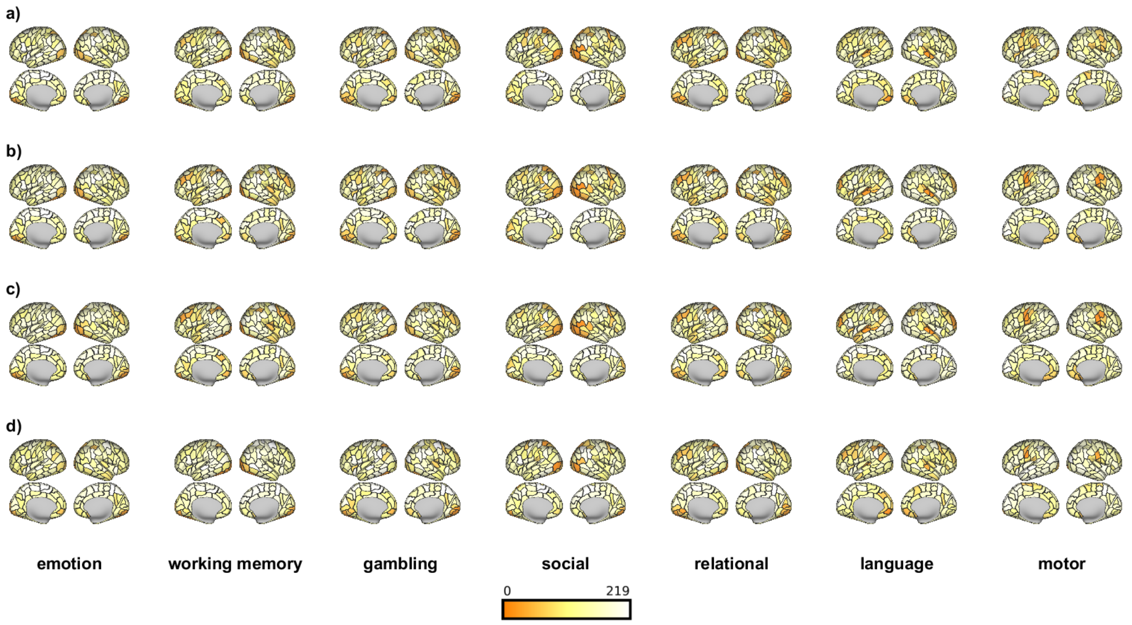

Supplemental Figure 1: **Results of rank ordering for regional control analyses per task and measure**.
Results of regional control analyses per task and measure. Columns display tasks, rows display measures. Plotted is a region's mean rank in regional control contribution across participants and tasks, with dark colors indicating low ranks and thus high control contribution, light colors indicating high ranks and thus low control contribution. Black borders indicate different regions as defined in the Lausanne atlas.
a) and b) show mean nodal rank across participants for stability of the stable and instable state, respectively
c) and d) show mean nodal rank across participants for control energy needed to move from the stable to instable and instable to stable state, respectively

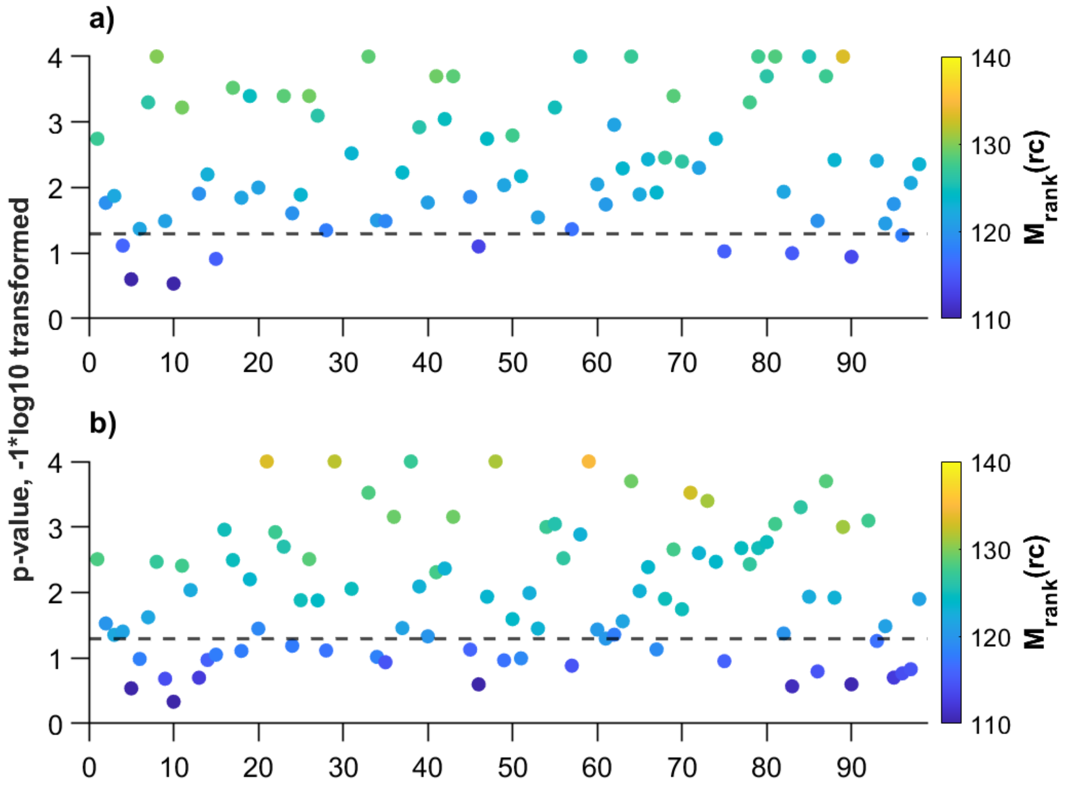

Supplemental Figure 2: **The rich club contributes significantly less to regional control than the periphery in the majority of participants**.
We repeated group-level evaluations of the mean regional control rank of the rich club on a participant level to account for interindividual variation in rich club constitution. The rich club was not significantly involved in control for any participant; for the majority of the sample, it was significantly not-involved. Shown are the log-transformed p-values of comparing the mean regional control rank of participant's rich club to the spin-test based null distribution. Smaller y-values indicate greater p-values. The dashed line indicates a p-value of 0.05; data points above the line show participants for which the individual rich club was significantly not involved in regional control; data points below the line show participants for which the individual rich club was not differentially involved in control than a size-matched set of random regions. Colors indicate the mean regional rank, and thus control contribution, of the rich club, with lower rank indicating higher control contributions.
a) Shows values across all tasks and stability measures; the rich club was significantly not involved in regional stability in 90.82% of participants.
b) Shows values across all tasks and energy measures; the rich club was significantly not involved in regional control of state transitions in 72.45% of participants.

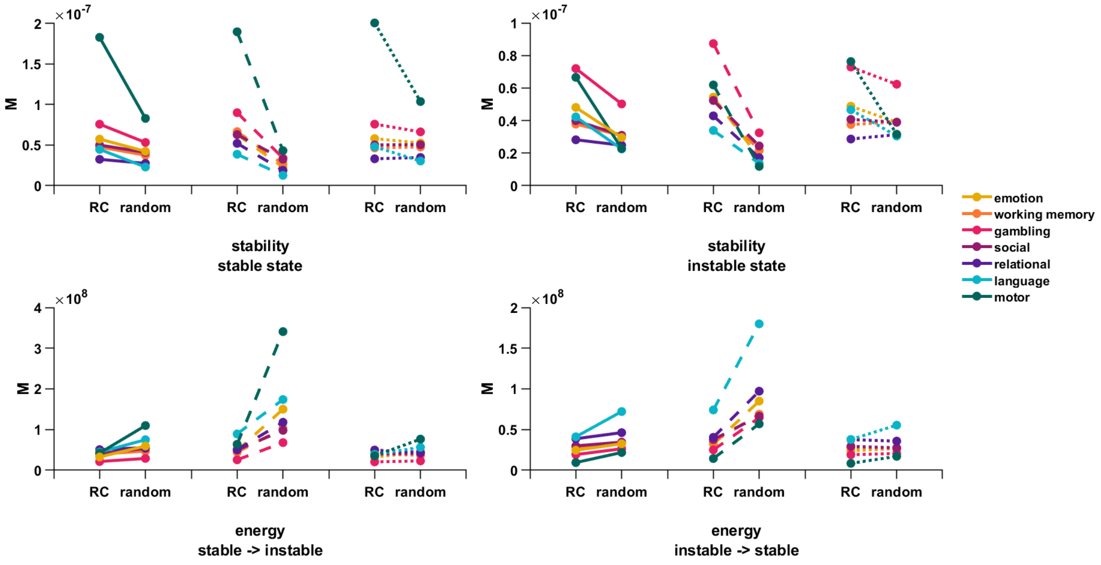

Supplemental Figure 3: **Main effect holds for FA-based and NOS-based connectomes and for a subset of measures in binary connectomes**.

Results of comparing exclusion of rich club (RC) and exclusion of a size-matched set of random regions when connectomes are average FA-based (solid line plots), NOS-based (dashed plots) or binary networks (dotted plots). Displayed are mean NCT values across individuals and for each measure.

M = mean value across participants, RC = rich club

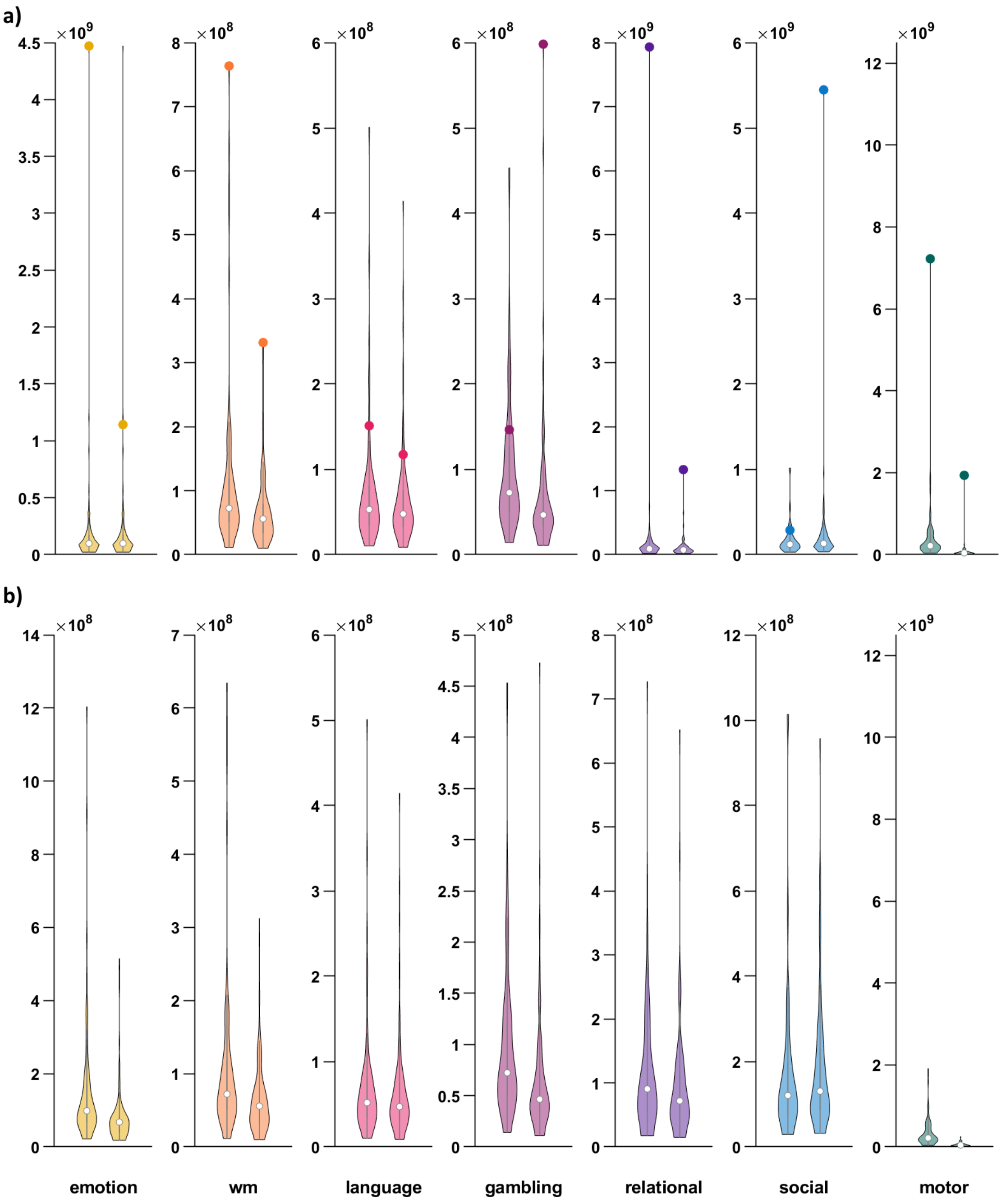

Supplemental Figure 4: **Distributions of NOS-based energy values when including vs. when excluding a major outlier participant**.
When using a NOS-based connectome for our analyses, we noticed that the control energy values of a single outlier participant distorted results. We here show violins comparing distributions when including the outlier (a) versus when excluding that participant from the sample (b). This outlier participant is excluded for NOS-based results reported in the main text. Shown are distributions of mean energy values for exclusion of randomly selected regions, across participants. For each task, the left plot corresponds to the mean energy values for the transition from the stable to instable state; and the right plot corresponds to the transition from the instable to stable state.
a) Distributions when including the outlier participant; values of the outlier participant are represented with colored dots. This specific individual was qualifying as an outlier in 10 out of 14 possible energy measures.
b) Distributions when the outlier participant shown above was removed from the sample. Statistical evaluations are based on this adjusted sample for NOS-based analyses; we additionally report results for the full sample in Supplemental Tables S11 and S12.

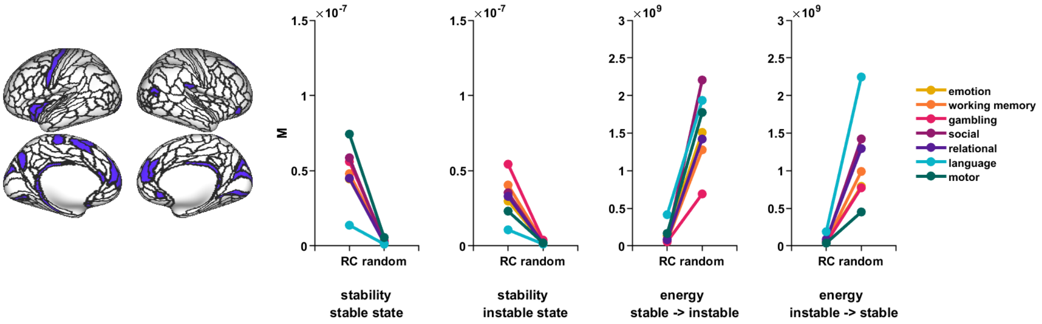

Supplemental Figure 5: **Replication of main finding a further widely used parcellation scheme**.

We were able to replicate our main results regarding a control role of the rich club based on an additional widely used parcellation scheme. As the figure shows, stability of a brain state always remained significantly higher when the rich club was prohibited from control compared to a semi-randomly selected set of reference regions; and energy needed to traverse between brain states overwhelmingly remained significantly lower. These findings indicate that the rich club was not significantly involved in optimal control for this additional parcellation scheme.

The figure shows group-level rich club nodes (blue) and analysis results for the 358-region multimodal parcellation scheme provided by the Human Connectome Project; two hippocampal nodes included in the original 360-region version were excluded from our analysis.

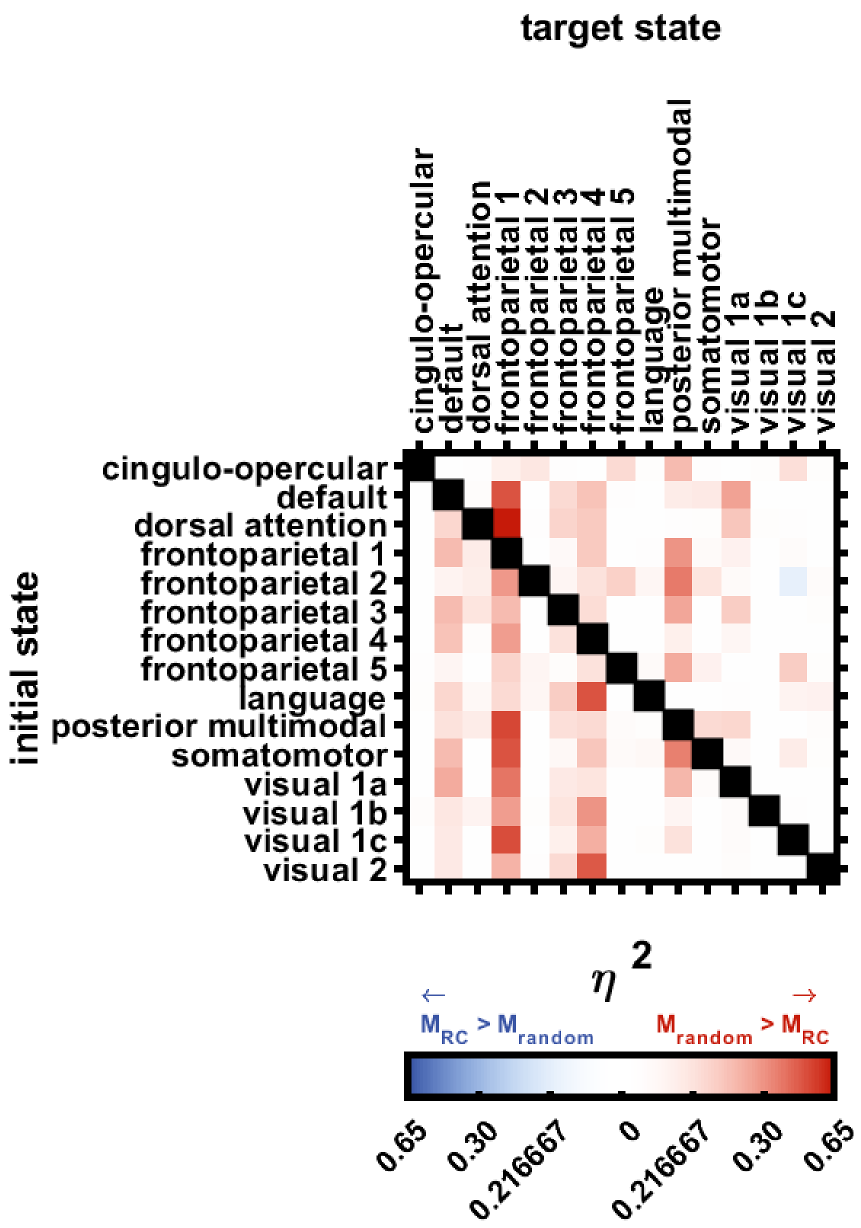

Supplemental Figure 6: **The rich club does not control brain state transitions across resting-state derived brain states; effect sizes depend on the target state**.
We used 15 independent functional components extracted from resting state data, as provided by the HCP, and replicated our main investigations regarding a control role of the rich club on these brain states. Shown are effect sizes for the difference in control metrics when excluding the rich club vs. when excluding random regions from control. We name the initial and target state according to the intrinsic connectivity networks they reflect. Red colors indicate a more drastic impact when excluding random regions, blue colors indicate a more drastic impact when excluding the rich club. The more saturated the color, the higher the effect size. Except for one state transition (blue square), the rich club overwhelmingly was not involved in control of transitions between various brain states derived from resting state data.
